# Self-Protection against Gliotoxin—A Component of the Gliotoxin Biosynthetic Cluster, GliT, Completely Protects *Aspergillus fumigatus* Against Exogenous Gliotoxin

**DOI:** 10.1371/journal.ppat.1000952

**Published:** 2010-06-10

**Authors:** Markus Schrettl, Stephen Carberry, Kevin Kavanagh, Hubertus Haas, Gary W. Jones, Jennifer O'Brien, Aine Nolan, John Stephens, Orla Fenelon, Sean Doyle

**Affiliations:** 1 Department of Biology and National Institute for Cellular Biotechnology, National University of Ireland Maynooth, Maynooth, Co. Kildare, Ireland; 2 Biocenter-Division of Molecular Biology, Innsbruck Medical University, Innsbruck, Austria; 3 Department of Chemistry, National University of Ireland Maynooth, Maynooth, Co. Kildare, Ireland; University of Wisconsin-Madison, United States of America

## Abstract

Gliotoxin, and other related molecules, are encoded by multi-gene clusters and biosynthesized by fungi using non-ribosomal biosynthetic mechanisms. Almost universally described in terms of its toxicity towards mammalian cells, gliotoxin has come to be considered as a component of the virulence arsenal of *Aspergillus fumigatus*. Here we show that deletion of a single gene, *gliT*, in the gliotoxin biosynthetic cluster of two *A. fumigatus* strains, rendered the organism highly sensitive to exogenous gliotoxin and completely disrupted gliotoxin secretion. Addition of glutathione to both *A. fumigatus* Δ*gliT* strains relieved gliotoxin inhibition. Moreover, expression of *gliT* appears to be independently regulated compared to all other cluster components and is up-regulated by exogenous gliotoxin presence, at both the transcript and protein level. Upon gliotoxin exposure, *gliT* is also expressed in *A. fumigatus* Δ*gliZ*, which cannot express any other genes in the gliotoxin biosynthetic cluster, indicating that *gliT* is primarily responsible for protecting this strain against exogenous gliotoxin. GliT exhibits a gliotoxin reductase activity up to 9 µM gliotoxin and appears to prevent irreversible depletion of intracellular glutathione stores by reduction of the oxidized form of gliotoxin. Cross-species resistance to exogenous gliotoxin is acquired by *A. nidulans* and *Saccharomyces cerevisiae*, respectively, when transformed with *gliT*. We hypothesise that the primary role of gliotoxin may be as an antioxidant and that in addition to GliT functionality, gliotoxin secretion may be a component of an auto-protective mechanism, deployed by *A. fumigatus* to protect itself against this potent biomolecule.

## Author Summary

The pathogenic fungus *Aspergillus fumigatus* causes disease in immunocompromised individuals such as cancer patients. The fungus makes a small molecule called gliotoxin which helps *A. fumigatus* bypass the immune system in ill people, and cause disease. Although a small molecule, gliotoxin biosynthesis is enabled by a complex series of enzymes, one of which is called GliT, in *A. fumigatus*. Amazingly, nobody has really considered that gliotoxin might be toxic to *A. fumigatus* itself. Here we show that absence of GliT makes *A. fumigatus* highly sensitive to added gliotoxin and inhibits fungal growth, both of which can be reversed by restoring GliT. Neither can the fungus make or release its own gliotoxin when GliT is missing. We also show that gliotoxin sensitivity can be totally overcome by adding glutathione, which is an important anti-oxidant within cells. We demonstrate that gliotoxin addition increases the production of GliT, and that GliT breaks the disulphide bond in gliotoxin which may be a step in the pathway for gliotoxin protection or release from *A. fumigatus*. We conclude that gliotoxin may mainly be involved in protecting *A. fumigatus* against oxidative stress and that it is an accidental toxin.

## Introduction

Gliotoxin, which has a molecular mass of 326 Da and is an epipolythiodioxopiperazine (ETP), contains a disulphide bridge of unknown origin and has been shown to play a significant role in enabling the virulence of *Aspergillus fumigatus* [1]–[3]. The cytotoxic activity of gliotoxin is generally mediated by direct inactivation of essential protein thiols [4] and by inhibition of the respiratory burst in neutrophils by disrupting NADPH oxidase assembly, thereby facilitating *in vivo* fungal dissemination [5], [6]. The enzymatic machinery responsible for gliotoxin biosynthesis, and metabolism, is encoded by a multi-gene cluster in *A. fumigatus* which is coordinately expressed during gliotoxin biosynthesis [7], [8]. This cluster encodes *gliP*, a bimodular nonribosomal peptide synthetase (NRPS) which has been conclusively shown to be responsible for the biosynthesis of a Phe-Ser dipeptide, a gliotoxin precursor, by gene disruption (Δ*gliP* mutant) [9]–[12]. In fact, disruption of *gliP* within the gliotoxin biosynthetic cluster has resulted in the effective inhibition of all cluster gene expression in a Δ*gliP* mutant [9]. A putative transporter, encoded by *gliA*, has been shown to facilitate gliotoxin efflux, and increased tolerance to exogenous gliotoxin, when expressed in *Leptosphaeria maculans* [13]. *sirA* is a *gliA* ortholog in this organism and *L. maculans* Δ*sirA* was more sensitive to exogenous gliotoxin and sirodesmin than wild-type, however restoration of *sirA* in the mutant led to greater tolerance towards these metabolites [13]. Bok *et al*. [14] have demonstrated that disruption of a fungal Zn(II)_2_-Cys(6) binuclear cluster domain transcription factor (*gliZ*) results in the complete inhibition of all gliotoxin cluster gene expression and effective diminution of gliotoxin production [14]. Although GliP has been shown to activate and condense L-Phe and L-Ser to form a precursor diketopiperazine moiety, no information relating to subsequent modification (e.g., thiolation) is available [9]–[12], [15] and it is also unclear if *A. fumigatus* might need to protect itself against potential gliotoxin cytotoxicity [13].

Interestingly, addition of gliotoxin (up to 5 µg/ml) to *A. fumigatus* Δ*gliP* resulted in the up-regulation of selected gene expression (*gliI*, *J*, *T* and *N*) within the *gli* cluster and Cramer *et al.* [9] noted complete activation of the gene cluster (except *gliP*) following gliotoxin exposure (20 µg/ml). However, exposure of wild-type *A. fumigatus* Af293 to gliotoxin (20 µg/ml), for 24 h, did not result in any significant alteration in gliotoxin cluster expression [9]. The biological significance of these observations is unclear, apart from implying a role for gliotoxin in the regulation of the *gli* cluster in the absence of gliotoxin production.

It has recently been demonstrated that gliotoxin and sporidesmin, also an ETP toxin containing a disulphide bridge, are both substrates and inactivators of glutaredoxin (Grx1) [16]. These authors also confirmed that the intact disulphide form of these ETP moieties was essential for Grx1 inactivation and that prior reduction of sporidesmin, using glutathione, prevented subsequent Grx1 inactivation. Oxygen presence was also required for Grx1 inactivation by sporidesmin and mass spectrometric analysis confirmed the formation of mixed disulphides between one molecule of Grx1 and either gliotoxin or sporidesmin, respectively. Combined, these data suggest interplay between oxygen availability and selective protein inactivation in the presence of oxidised ETP-type molecules. This indirectly suggests either a protective, or neutral, involvement of the oxidised forms of gliotoxin or sporidesmin in protecting against the deleterious effects of oxygen by selective protein inactivation.

In mammalian cells it has been demonstrated that the oxidized form of gliotoxin is actively concentrated in a glutathione-dependent manner and that it then exists within the cell almost exclusively in the reduced form [17]. As glutathione levels fall due to apoptosis, the oxidized form of gliotoxin effluxes from the cell where the cytocidal effects of gliotoxin are perpetuated in a pseudocatalytic manner. Conversely, it has been shown that gliotoxin may substitute for 2-cys peroxiredoxin activity in HeLa cells by accepting electrons from NADPH via the thioredoxin reductase–thioredoxin redox system to reduce H_2_O_2_ to H_2_O. In this way, nanomolar levels of gliotoxin may actually protect against intracellular oxidative stress [18].

Although the cytotoxic effects of gliotoxin on mammalian cells have been extensively investigated, and yeast have been deployed as a model system to study this interaction [19], no direct investigation of any self-protective mechanism used by *A. fumigatus* against this intriguing molecule has been undertaken. Here, we demonstrate that deletion of *gliT* results in transformants which cannot grow in the presence of even modest levels of exogenous gliotoxin and that exogenous gliotoxin up-regulates gene expression within the gliotoxin cluster, especially that of *gliT*. We propose that GliT is the key cellular defence against gliotoxin in *A. fumigatus* and that this finding yields a new selection marker system for detecting transformation.

## Results

### Deletion and complementation of *gliT* in *A. fumigatus*

Δ*gliT* mutants were generated by transformation of *A. fumigatus* strains ATCC46645 and ATCC26933, respectively, as described in Materials and Methods, using the bipartite marker technique and pyrithiamine selection, with modifications [20], [21] (Figure S1). Deposition number: IMI CC 396691 (CABI Bioscience Centre, Egham, Surrey, UK). These two strains were chosen because ATCC26933 is a gliotoxin producer, whereas ATCC46645 lacks significant gliotoxin production using the Minimal Media described in Materials and Methods (see below). Complementation of *gliT* mutant strains was carried out as described in Materials and Methods and Figure S1). Complemented strains (*gliT* ^C^) (Deposition number: IMI CC 396692) exhibited wild-type like features in all subsequent experiments, demonstrating that the occurrence of a single ectopic integration of a *gliT* fragment is insignificant in the *A. fumigatus* ATCC26933 background.

### Gliotoxin prevents growth of Δ*gliT* strains

**Figure 1:**
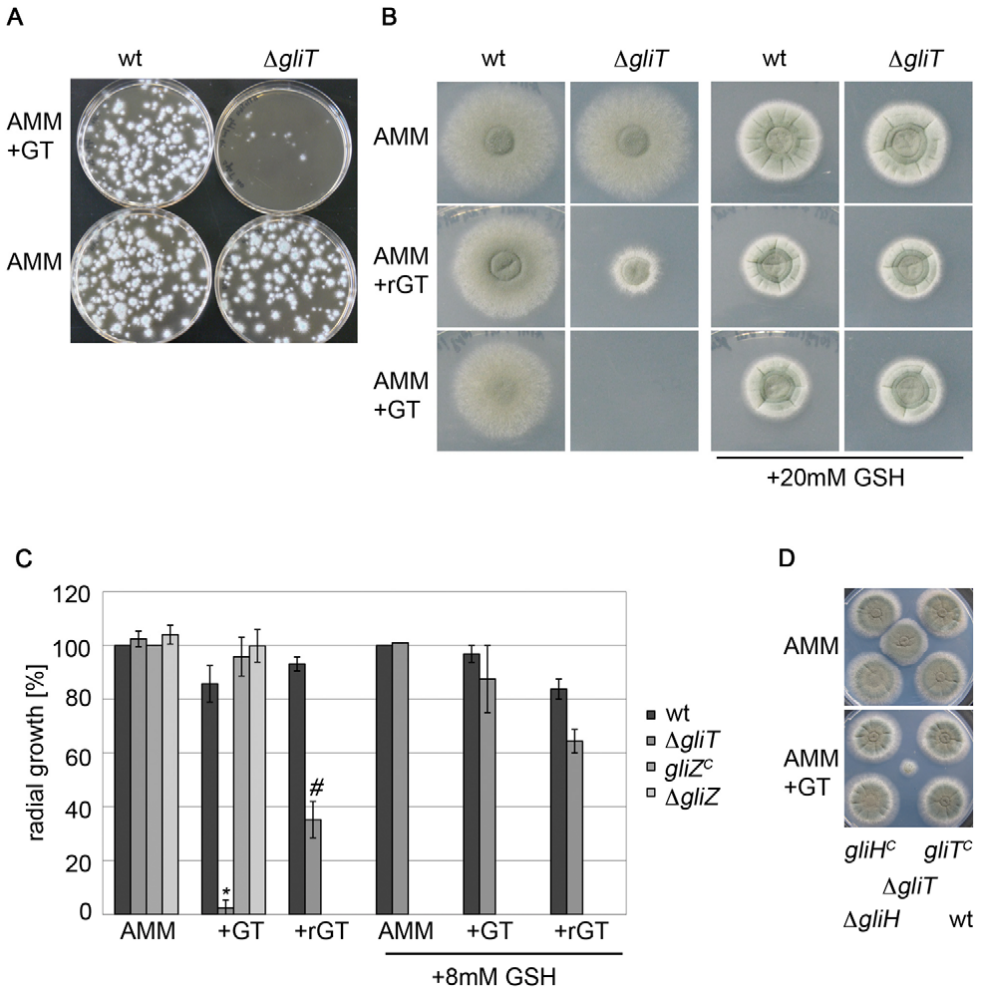
Exogenous gliotoxin specifically inhibits growth of *A. fumigatus* Δ*gliT*. (A) Protoplasts of *A. fumigatus* wild-type (ATCC46645) and Δ*gliT* were poured onto AMM plates, in the presence or absence of gliotoxin (10 µg/ml). Plates were incubated for 48 h at 37°C. (B) Conidia (*A. fumigatus* ATCC26933 and Δ*gliT;* 10^4^/spot) were dotted on AMM plates containing the indicated supplement and incubated for 48 h at 37°C. To obtain reduced gliotoxin (rGT) free of additional thiols, 10 µg/ml GT was reduced with 50 mM NaBH_4_ for 60 min at room temperature. Gliotoxin (GT) and rGT were added at a final concentration of 10 µg/ml. (C) Quantification of radial growth of *A. fumigatus* ATCC26933, Δ*gliT*, Δ*gliZ* and complemented Δ*gliZ* (*gliZ* ^c^) [14] in the presence of gliotoxin and rGT, with and without exogenous GSH. Strains (10^4^ conidia) were dotted on AMM containing GT (gliotoxin; 10 µg/ml), rGT (10 µg/ml), or reduced glutathione (8 mM), respectively. Colony diameter was measured after 48 h of incubation at 37°C and experiments were repeated in triplicate. * indicates a significance level of p<0.0001 and # indicates p<0.05. Note: For clarity, *A. fumigatus* Δ*gliZ* and *gliZ* ^c^ (*gliZ* complemented) data are only shown for AMM +/− GT only, as their growth was unaffected by all conditions tested. (D) *A. fumigatus* Δ*gliH* is unaffected by gliotoxin presence. 10^4^ conidia were spotted on AMM with GT (10 µg/ml, bottom) or without GT (top). Plates were incubated for 72 h (hence the visible background growth of ΔgliT). *A. fumigatus* Δ*gliH* did not show any sign of an altered growth phenotype in the presence of gliotoxin.

Δ*gliT* protoplasts grew and regenerated mycelia perfectly in the absence of gliotoxin (Figure 1A). The Δ*gliT* strain grew at identical rates to wild-type (data not shown). However, Δ*gliT* protoplasts were unable to grow in the presence of gliotoxin (10 µg/ml) (Figure 1A) whereas exogenous gliotoxin had no effect on wild-type growth. Subsequent phenotypic analysis of *A. fumigatus* ATCC46645, ATCC26933, and respective Δ*gliT* conidia (Δ*gliT* ^46645^ and Δ*gliT* ^26933^) demonstrated that gliotoxin (5 µg/ml) significantly inhibited Δ*gliT* growth on minimal medium and completely inhibited Δ*gliT* growth on both AMM and Sabouraud medium (gliotoxin, 10 µg/ml) (Figure 1B & C; *p*<0.0001 and Figure S2). Moreover, germination rates of Δ*gliT* strains were comparable to those of wild-type *A. fumigatus*, even in the presence of gliotoxin up to 10 µg/ml. These results clearly indicated that Δ*gliT* was highly sensitive to exogenous gliotoxin. Consequently, Δ*gliT* ^46645^ and Δ*gliT* ^26933^ mutant complementation was carried out by introducing *gliT* only (no antibiotic resistance gene) to complement Δ*gliT* with selection in the presence of gliotoxin (10 µg/ml). Transformants, which had recovered resistance to exogenous gliotoxin, were confirmed by Southern analysis to have an intact and functional copy of *gliT* present (Figure S1). This result confirms that *gliT* confers resistance to gliotoxin in *A. fumigatus* and that Δ*gliT* mutants have significant potential for future functional genomic studies involving *A. fumigatus* since gene deletions in this strain are selectable by *gliT* reintroduction, with selection in the presence of gliotoxin.

Remarkably, addition of reduced glutathione (GSH; 20 mM)) to test plates completely abolished the cytotoxic effects of exogenous gliotoxin which indicated that *gliT* loss resulted in depletion of intracellular GSH, when exposed to gliotoxin, or that only the oxidized form of gliotoxin is imported into *A. fumigatus* (Figure 1B & C). Prior reduction of gliotoxin, using 50 mM NaBH_4_, resulted in a statistically significant inhibitory effect of gliotoxin on growth of Δ*gliT* ^26933^ (*p*<0.05) (Figure 1B & C). NaBH_4_ was selected as reductant as it avoided complications associated with the introduction of additional thiols, or GSH, and the formation of gliotoxin conjugates, which may have resulted from GSH, DTT or β-mercaptoethanol-mediated reduction. It was also observed that GSH presence (8 mM) partially alleviated the growth inhibitory effects of gliotoxin (with or without prior reduction; p<0.01 and p<0.005, respectively) (Figure 1C). However, wild-type levels of growth were only achieved in the presence of 20 mM GSH (Figure 1B). The enhanced GSH-mediated alleviation of gliotoxin-induced cytostatic effects observed in Δ*gliT*, strongly suggest that depletion of intracellular glutathione may be a consequence of *gliT* loss. GSH-mediated relief of *A. fumigatus* Δ*gliT* growth inhibition, by exogenous NaBH_4_-reduced gliotoxin, indicates that intracellular GSH depletion plays a role in the inhibitory effect of gliotoxin- and not that GSH is merely acting to reduce exogenously added gliotoxin and prevent uptake (Figure 1B & C). Exogenous gliotoxin or reduced gliotoxin had no effect on growth of Δ*gliZ* and *gliZ* ^c^ (*gliZ* complemented strain) [14] (kind gifts from Professor Nancy Keller, University of Wisconsin-Madison) and an identical pattern was observed in the presence of GSH (data not shown). Moreover, *A. fumigatus* Δ*gliT* did not exhibit any phenotype when exposed to either H_2_O_2_ or phleomycin (data not shown). *A. fumigatus gliT* ^C^ strains were resistant to exogenous gliotoxin (Figure 1D).

### Gliotoxin induces expression of the gliotoxin gene cluster

**Figure 2:**
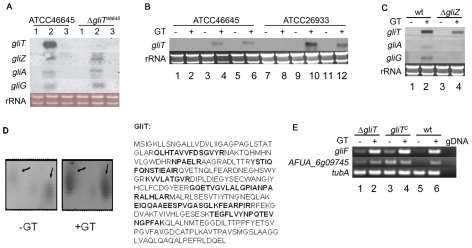
*gliT* expression. (A) Northern analysis of the induction of gliotoxin gene cluster expression in *A. fumigatus* ATCC46645 and Δ*gliT*. Lanes 1, 2 and 3 correspond to *A. fumigatus* RNA extracts from 21 h AMM, 21 h AMM shifted to gliotoxin (5 µg/ml) for 3 h and 24 h AMM, respectively. (B) Gliotoxin induction of *gliT* expression in *A. fumigatus gliT* ^C^ strains. Lanes 1–6 and 7–12 contain RNA from strains in the ATCC46645 and ATCC26933 backgrounds, respectively. Lanes 1 and 7: *A. fumigatus* Δ*gliT* 24 h AMM; Lanes 2 and 8: *A. fumigatus* Δ*gliT* 21 h AMM+3 h gliotoxin (5 µg/ml); Lane 3 and 9: *A. fumigatus gliT* ^C^ 24 h AMM; Lanes 4 and 10: *A. fumigatus gliT* ^C^ 21 h AMM+3 h gliotoxin (5 µg/ml); Lanes 5 and 11: *A. fumigatus* wt 24 h AMM; Lane 6 and 12: *A. fumigatus* wt 21 h AMM+3 h gliotoxin (5 µg/ml). (C) Expression of *gliT* in Δ*gliZ* following exposure to gliotoxin. Cultures of *A. fumigatus* ATCC46645 (lanes 1 and 2) and Δ*gliZ* (lanes 3 and 4) were grown for 24 h in AMM (Lane 1 and 3) or pulsed with gliotoxin (5 µg/ml) after 21 h and cultured for a further 3 h (Lane 2 and 4). Although gliotoxin induced expression of *gliA* and *gliG* in wild-type, neither *gliA* or *gliG* are expressed in Δ*gliZ*. All Northern analyses were performed with 10 µg of total RNA isolated from strains grown in AMM for 24 h with or without gliotoxin. (D) *A. fumigatus* GliT expression and identification. Quantitative 2D-PAGE analysis confirmed increased expression (threefold) of GliT following exogenous gliotoxin (GT) addition to *A. fumigatus* cultures (GliT appears to exist as two isoforms of different *pI* (5.5–5.6) and M*r*). Peptides identified by MALDI-ToF mass spectrometry are highlighted in bold (33% sequence coverage) and mass spectrum is given in Figure S3. (E) Semi-quantitative RT-PCR of *gliT* adjacent genes in *A. fumigatus* wild-type (wt) (ATCC26933) and isogenic mutant strains. Expression of *gliF* and AFUA_6g09745 (*gliH*) was examined. As a control *tubA* expression was monitored. As a negative control genomic DNA (gDNA) was used as template. Lane 1: *A. fumigatus* Δ*gliT* ^26933^ 24 h AMM. Lane 2: *A. fumigatus* Δ*gliT* ^26933^ 21 h AMM+3 h gliotoxin (5 µg/ml). Lane 3: *A. fumigatus gliT* ^C^ 24 h AMM. Lane 4: *A. fumigatus gliT* ^C^ 21 h AMM+3 h gliotoxin (5 µg/ml). Lane 5: *A. fumigatus* wt 24 h AMM. Lane 6: *A. fumigatus* wt 21 h AMM+3 h gliotoxin (5 µg/ml).

*gliZ*, *A* and *G* encode the gliotoxin cluster transcription factor, transporter and a putative glutathione s-transferase (generally a detoxification enzyme), respectively, and all are conceivably involved in protection against gliotoxin toxicity [3], [8], [22]. Northern analysis showed that expression of these 3 genes plus *gliT*, from the gliotoxin gene cluster, was induced in *A. fumigatus* ATCC46645 within 3 h following gliotoxin (5 µg/ml) addition at 21 h (Figure 2A). No *gliT* expression was detectable in Δ*gliT* whereas the expression of all other genes was identical to the wild-type, including the continued absence of expression at 24 h in the absence of added gliotoxin (Figure 2A). Expression of *gliT* was restored in pyrithiamine-resistant *A. fumigatus gliT* ^C^ derived from both ATCC46645 and ATCC26933 strain backgrounds, which unambiguously confirms restoration of *gliT* expression in complemented strains (Figure 2B). Moreover, *gliT* expression was inducible by addition of gliotoxin (5 µg/ml), as had been observed in both wild-type strains, thereby convincingly demonstrating that the wild-type phenotype had been entirely restored (Figure 2B). As noted above, no significant growth inhibition of *A. fumigatus* Δ*gliZ* in particular, or *gliZ* ^c^, was observed in the presence of gliotoxin or reduced gliotoxin (Figure 1C). These observations further confirm the minimal role played by any other component of the *gli* gene cluster in protection against gliotoxin presence since *gliZ* absence results in complete cluster attenuation [14]. Significantly, Northern analysis confirmed gliotoxin-induced *gliT* expression in Δ*gliZ*, which indicates the independent regulation of *gliT* with respect to other *gli* cluster components, such as *gliG* and *gliA* which are not expressed by *A. fumigatus* Δ*gliZ* following exposure to gliotoxin (Figure 2C). These observations are in complete accordance with proteomic data which demonstrated a threefold up-regulation of GliT expression (33% sequence coverage) in *A. fumigatus* ATCC26933, and the absence of detection of any other *gli* cluster component, following 3 h exposure to exogenous gliotoxin (14 µg/ml) (Figure 2D and Figure S3).

### Genes immediately adjacent to *gliT* in the gliotoxin gene cluster do not mediate resistance to exogenous gliotoxin

Sequence analysis of the 5′ and 3′ regions adjacent to the original *gliT* locus in *A. fumigatus* Δ*gliT* ^26933^ confirmed that *gliF* was intact but revealed two mutations (C23R and E160G) in the open reading frame of a gene (AFUA_6G09745; identified as a conserved hypothetical protein at http://www.cadre-genomes.org.uk (but here termed *gliH*), located 3′ with respect to the *gliT* locus. Although expression of *gliF* and *gliH* was confirmed by RT-PCR in *A. fumigatus* Δ*gliT* ^26933^ (Figure 2E), there was concern that the altered sequence of *gliH* may have resulted in a mutant enzyme, which could possibly have also contributed to gliotoxin sensitivity in Δ*gliT* ^26933^. However, *A. fumigatus* Δ*gliH* ^26933^ grew in the presence of gliotoxin (10 µg/ml) (Figure 1D) which completely eliminated the possibility that this gene, located adjacent to *gliT* in the *A. fumigatus* genome, contributed to gliotoxin resistance and established, beyond question, the key role of *gliT* in mediating resistance to exogenous gliotoxin. *A. fumigatus gliH* ^C^ (Figure S1) was also resistant to exogenous gliotoxin, as expected (Figure 1D).

### Gliotoxin is not produced by *A. fumigatus* Δ*gliT*

**Figure 3:**
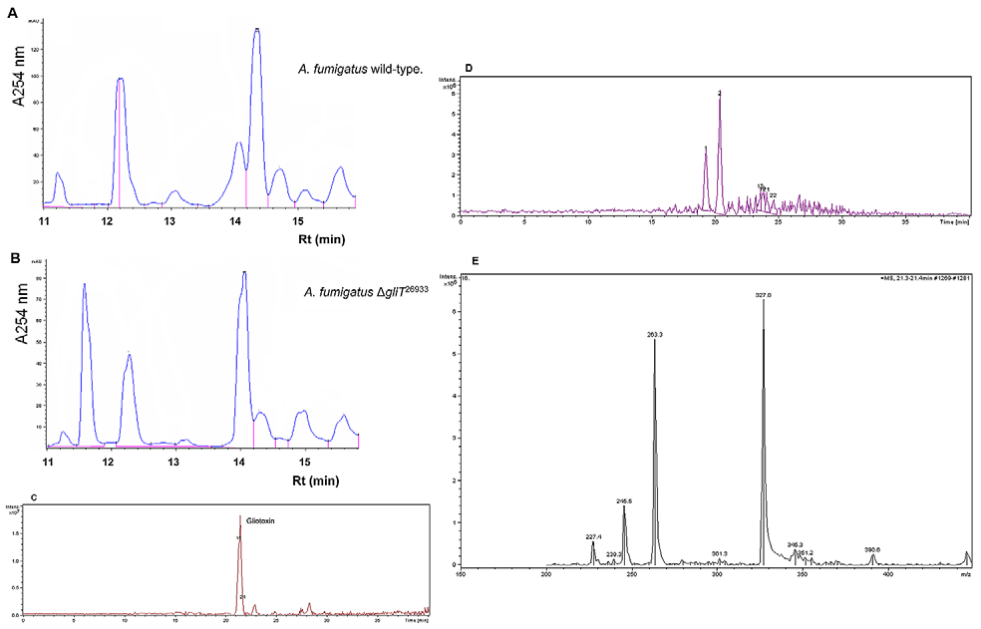
Gliotoxin is not produced by *A. fumigatus* Δ*gliT*. (A) HPLC chromatogram of an organic extract from *A. fumigatus* ATCC26933 indicating gliotoxin presence with Rt = 14.5 min. (B) HPLC chromatogram of organic extracts from *A. fumigatus* Δ*gliT* ^26933^ indicating gliotoxin absence but a new metabolite with Rt = 11.7 min. (C) LC-MS analysis of an organic extract from *A. fumigatus* ATCC26933 indicating gliotoxin presence. (D) LC-MS analysis of *A. fumigatus* Δ*gliT* ^26933^ confirms absence of secreted gliotoxin. (E) MS^2^ of gliotoxin present in wild-type *A. fumigatus* indicating expected sub-fragments as noted previously [10].

Gliotoxin (580 ng/ml) was detectable in organic extracts from *A. fumigatus* ATCC26933 but not Δ*gliT* ^26933^ cultures, grown under identical conditions, by RP-HPLC and LC-MS analysis (Figure 3). Gliotoxin production was recovered in *A. fumigatus* ATCC26933 *gliT* ^C^ (Figure S4) Interestingly, Δ*gliT* ^26933^ exhibited an identical phenotype to Δ*gliT* ^46645^ which was generated from *A. fumigatus* ATCC46645, yet gliotoxin production was undetectable, under the culture conditions employed, in both *A. fumigatus* ATCC46645 and Δ*gliT* ^46645^ indicating that sensitivity to exogenous gliotoxin is not associated with a *de novo* gliotoxin biosynthetic capacity. A metabolite with retention time (Rt) = 11.7 min (A_220 nm_) was apparent in Δ*gliT* ^26933^ extracts which was absent in wild-type extracts (Figure 3). This material was purified to assess any growth inhibitory effect, however when added to AMM cultures of Δ*gliT* or wild-type no alteration of growth rates was observed (data not shown). High resolution LC-ToF MS analysis of the metabolite (from Figure 3B) confirmed the presence of a molecular ion with a mass of 279.0796 *m/z* ((M+H)^+^) (Figure S4). This accurate mass value (279.0796 *m/z*) corresponded to a predicted molecular formula of C_13_H_15_N_2_O_3_S for the ion whereby the calculated exact mass for C_13_H_15_N_2_O_3_S + H^+^ was 279.0798 Da using Agilent Technologies Masshunter workstation software. This result suggests that a monothiol form of gliotoxin could have been secreted from *A. fumigatus* Δ*gliT* ^26933^. A molecular species of m/z 279, which yielded daughter ions of m/z 261.1, 231.0 and 203.1, upon MS^2^ analysis, was also detected by LC-MS analysis of the purified gliotoxin-related metabolite from *A. fumigatus* Δ*gliT* ^26933^ (Figure S4). Gliotoxin was not produced by *A. fumigatus* Δ*gliH* ^26933^ (Figure S4) which strongly supports a role for this gene in gliotoxin biosynthesis or secretion, but not protection against exogenous gliotoxin. This result was further consolidated whereby no gliotoxin production was detectable, by HPLC-DAD or LC-MS, in *A. fumigatus* Δ*gliT^26933gliH^* (data not shown), which was generated by restoration of the fully intact *gliH* in *gliT*-deficient *A. fumigatus* (Figure S1).

### GliT exhibits a gliotoxin reductase activity

**Figure 4:**
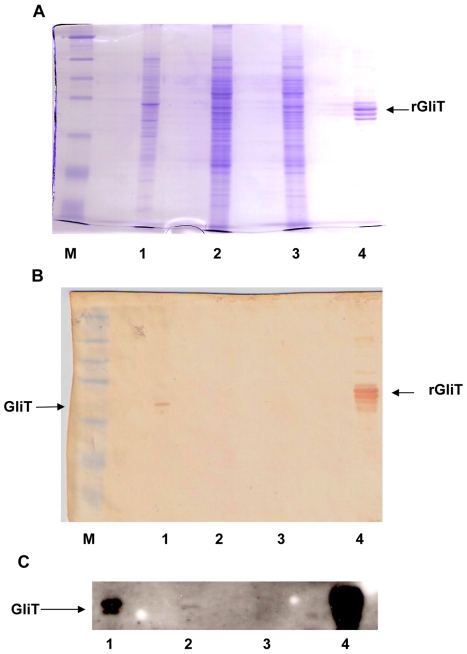
Immunoaffinity purified human IgG detects native GliT in *A. fumigatus*. (A) SDS-PAGE and (B/C): Western blot analysis of *A. fumigatus* cell lysates. Immuno-affinity purified human IgG[anti-GliT] was used for Western analysis followed by anti-human IgG-HRP conjugate with visualization by either (B) diaminobenzidine or (C) ECL detection. Lane M: Mr marker: Lane 1: *A. fumigatus* ATCC26933 lysate (72 h culture); Lane 2: *A. fumigatus* ATCC46645 lysate (24 h culture); Lane 3: *A. fumigatus* ATCC46645 Δ*gliT* lysate (24 h culture) and Lane 4: Recombinant GliT (2 µg). Immunoaffinity purified human IgG to GliT identified GliT in all except *A. fumigatus* Δ*gliT*, however ECL substrate was required to detect low level GliT expression in *A. fumigatus* ATCC46645 (lane C.2).

Recombinant GliT was expressed in, and purified by differential extraction from, *E.coli* with a yield of approximately 5.7 mg per gram of cells. However the protein was completely insoluble and was refractory to any attempts at refolding for activity analysis (data not shown). SDS-PAGE analysis confirmed a subunit molecular mass of 38 kDa for recombinant GliT (Figure S5), which appears to migrate as a dimer under non-reducing conditions (Figure S5), and protein identity was unambiguously confirmed by MALDI-ToF MS whereby peptides (following tryptic digestion) were identified yielding 21% sequence coverage (Figure S6). Immunoaffinity purification of GliT-specific human IgG was achieved by incubation of human sera with Sepharose-coupled recombinant GliT. The specificity of this GliT-specific human IgG was confirmed by the successful detection of native GliT in both *A. fumigatus* cell lysates, and partially-purified extracts of *A. fumigatus* (Protocol S1; Figure 4). Notably, GliT was not detectable in *A. fumigatus* Δ*gliT* (Figure 4).

**Figure 5:**
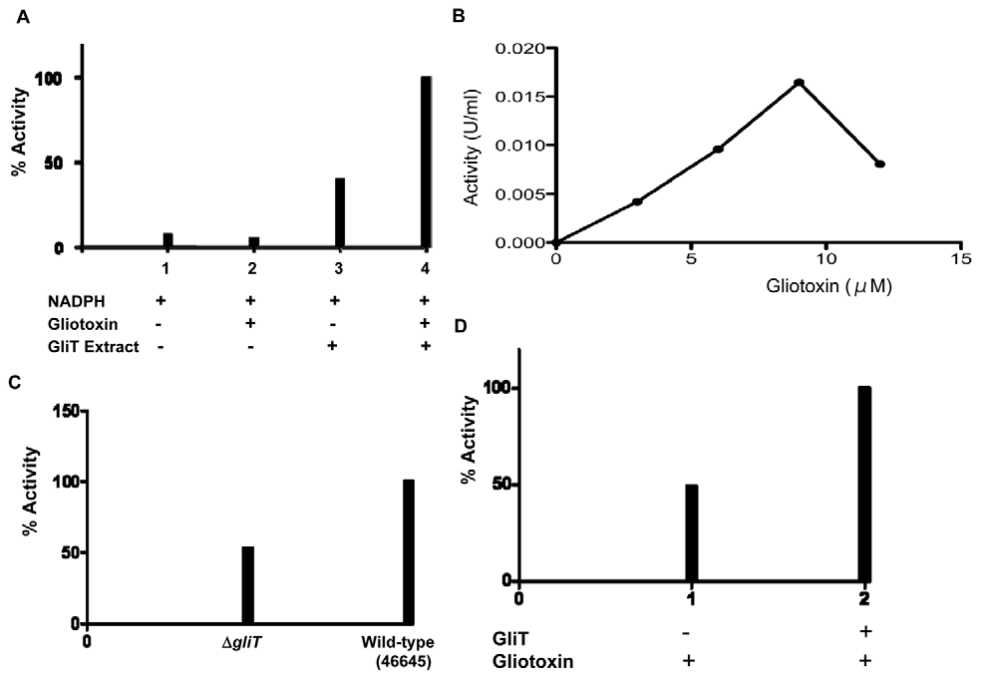
GliT exhibits a gliotoxin reductase activity. (A) No GliT activity (gliotoxin reductase) is detectable in the absence of gliotoxin or GliT (1 & 2). Background NADPH oxidase activity is detectable in semi-purified *A. fumigatu*s cell extracts (3) (Figure S5), however, GliT-mediated gliotoxin reductase activity is detectable upon addition of gliotoxin (4). (B) *In vitro*, optimal GliT gliotoxin reductase activity is observed up to 9 µM gliotoxin. This activity is inhibited at higher gliotoxin concentrations (>12 µM). (C) Relative gliotoxin reductase activity in cell lysates from Δ*gliT* compared to *A. fumigatus* ATCC46645 with gliotoxin addition during culture. Wild-type lysates exhibit enhanced gliotoxin reductase activity (47%) consequent to elevated GliT expression. (D) Immunodepletion of GliT from semi-purified *A. fumigatu*s cell extracts (Figure S5), using immunoaffinity purified human IgG [anti-GliT], results in a 51% decrease in gliotoxin reductase activity.

Previous hypotheses have suggested that GliT may only exhibit gliotoxin oxidase activity (responsible to disulphide bridge closure during biosynthesis) (3, 8, 22). However, following gliotoxin induction of *A. fumigatus* ATCC46645, enhanced GliT activity was evident in cell lysates and native GliT was partially purified by ammonium sulphate precipitation and ion-exchange chromatography (Figure S7). Data presented in Figure 5A confirm that partially-purified native GliT specifically catalyses the NADPH-mediated reduction of oxidized gliotoxin, whereby NADPH oxidation is only evident in the presence of both gliotoxin (9 µM) and GliT-containing lysates. Hence, GliT appears to exhibit gliotoxin reductase activity which can catalyse disulphide bridge cleavage, at concentrations up to 9 µM gliotoxin (Figure 5B). This activity is inhibited at higher gliotoxin concentrations (>12 µM). Not unexpectedly, *A. fumigatus* cell extracts appear to contain basal NADPH oxidase activity which yields background, non-specific NADPH oxidation (Figure 5A). Thus, *A. fumigatus* ATCC46645 and Δ*gliT* lysates, generated without prior gliotoxin induction of GliT expression, exhibit near-identical activity. However, significantly greater gliotoxin reductase activity (2:1) was apparent in *A. fumigatus* ATCC46645, than Δ*gliT*, cell lysates following gliotoxin exposure (Figure 5C). Immunoprecipitation of GliT from partially purified *A. fumigatus* cell lysates (Figure S7) using human IgG [anti-GliT] resulted in a 51% reduction of gliotoxin reductase (NADPH oxidase) activity (Figure 5D), in complete accordance with data in Figure 5C, further confirming enzyme specificity. Interestingly, GliT activity was not enhanced in the presence of thioredoxin from *Spirulina* sp., in activity assays, which indicates that GliT is specific for gliotoxin reduction and that it may operate independently of cellular thioredoxin reductase/thioredoxin systems.

Expression of GliT in *A. fumigatus* was further explored by fluorescence confocal microscopy. Data in Figure S8A-C confirm transformation of *A. fumigatus* Δ*gliT* ^46645^ and that *gliT-gfp* expression is enhanced by gliotoxin addition. As shown in Figure S8A, it appears that low-level GliT expression is evident throughout mycelia without gliotoxin addition. However, following mycelial exposure to gliotoxin (5 µg/ml), an enhancement of GliT expression in the cytoplasm, and in nuclei, as shown by fluorescence intensities (Figure S8B & C), is observed - which is in complete agreement with proteomic, molecular and enzyme activity observations. Expression of GliT-GFP fusion protein completely restored gliotoxin resistance (10 µg/ml), although colonies appeared white (Figure S9).

The concordance of these data lead us to conclude that a GliT-mediated gliotoxin reductase activity is induced by exposure of *A. fumigatus* to gliotoxin.

### GliT is not required for *A. fumigatus* virulence in *Galleria mellonella*

A prerequisite for testing *A. fumigatus* Δ*gliT* virulence was to evaluate the utility of our *G. mellonella* infection model. To this end, assessment of the relative virulence of *A. fumigatus* Δ*gliZ* and corresponding wild-type in *G. mellonella*, in either the presence or absence of added gliotoxin, was assessed (Figure S10). Here, all *Galleria* exposed to *A. fumigatus* Δ*gliZ* were alive at 24 h and the wild-type strain exhibited greater virulence than Δ*gliZ* (60% (12/20) versus 20% (4/20) mortality, respectively), at 48 h post-inoculation, thereby confirming the utility of the model system for detection of alteration in virulence associated with gliotoxin production. To assess now the relative contribution of *gliT* to virulence of *A. fumigatus* we compared the survival of larvae of the greater wax moth *G. mellonella* following infection with 10^6^ conidia/larvae of *A. fumigatus* ATCC26933 and *gliT* ^C^ to that of larvae (*n* = 20) infected with the same dose of Δ*gliT* ^26933^ (Figure S10). For all groups of infected larvae, 100% mortality was recorded after 72 h and the degree of melanisation was not distinguishable between these groups. Also, pretreatment of larvae with gliotoxin (0.5 µg/larva in 20 µl) did not lead to an attenuation of virulence of Δ*gliT* (Figure S10). Notably, similar results were obtained using ATCC46645 and Δ*gliT^46645^* strains (data not shown). These results clearly show that, *gliT* has a minimal, if any, role to play in the virulence of *A. fumigatus* employing a Galleria model.

### GliT confers protection against exogenous gliotoxin in *Aspergillus nidulans* and *Saccharomyces cerevisiae*

**Figure 6:**
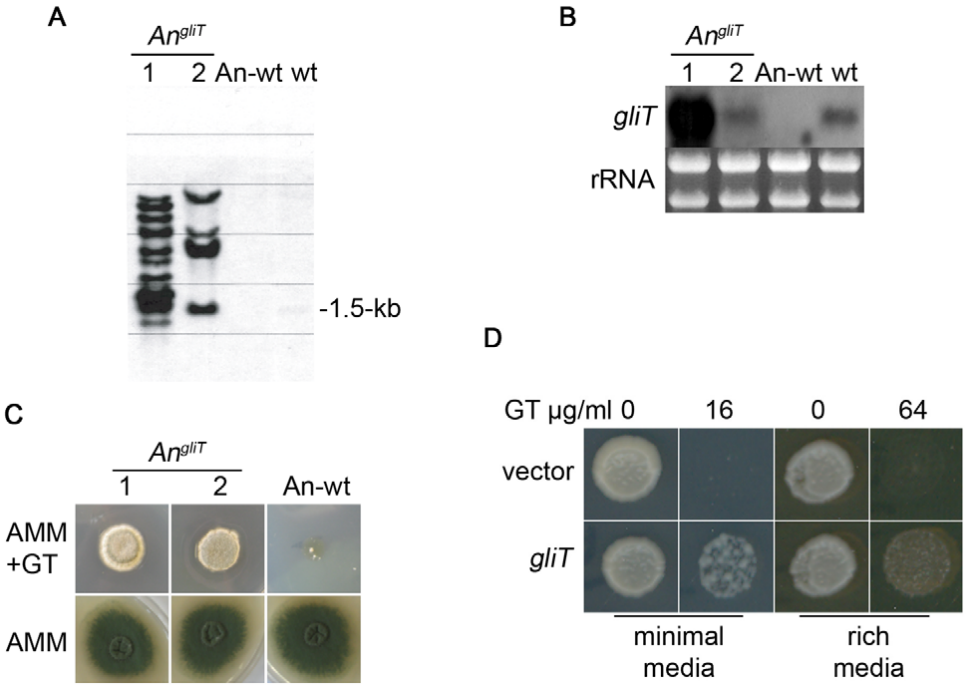
Transformation of *A. nidulans* and *S. cerevisiae* with *gliT* facilitates resistance to exogenous gliotoxin. (A) Southern analysis confirms that two *A. nidulans* transformants contain *gliT* (*An^gliT^* 7 and 8) compared to *A. nidulans* wild-type (An-wt). wt: *A. fumigatus* (positive control for *gliT*). Genomic DNA was digested with *Pst*I and probed for the presence of *A. fumigatus gliT*. (B) Northern analysis of *gliT* in An-wt, *An^gliT^* (7 and 8) and *A. fumigatus* wild-type (wt). RNA was isolated using TRI-reagent and 10 µg of total RNA were probed with a *gliT*-specific probe. (C) Plate assay of *A. nidulans* wild-type and *A. nidulans* expressing *gliT* (*An^gliT^* 7 and 8). *A. nidulans* wild-type and *An^gliT^* (10^4^) conidia were dotted on AMM and on AMM containing gliotoxin (50 µg/ml). Growth was monitored over a period of 72 h at 37°C. Genotypes of strains are described in Table 1. (D) GliT confers gliotoxin resistance on *S. cerevisiae*. Spots represent equal numbers of yeast cells plated onto medium containing gliotoxin at the concentrations indicated. Plates were incubated at 30°C for two days followed by a further three days at room temperature.

Reintroduction of *gliT* into *A. fumigatus* Δ*gliT* was selected for in the presence of gliotoxin and no additional selection marker was required (Figure S1 and Figure 1D). To further test the ability of *gliT* to confer resistance to gliotoxin, and its future potential as a selection marker gene, we introduced *gliT* into *A. nidulans* which does not produce gliotoxin and neither does it contain any genes involved in gliotoxin biosynthesis [22], [23]. The absence of *gliT*, and cognate gene expression, in *A. nidulans* was confirmed by Southern and Northern analysis (Figure 6A & B). Subsequent transformation of *A. nidulans* with *A. fumigatus*-derived *gliT* resulted in the generation of three transformants (*An^gliT^* 1, 2 and 3) (Deposition number: IMI CC 396693), which were shown by Northern analysis to express *gliT* to different extents (Figure 6B). This led to acquisition of resistance to high levels of exogenous gliotoxin (50 µg/ml) (Figure 6C) thereby confirming the key role of *gliT* in protection against gliotoxin toxicity in gliotoxin-naïve fungi. The *gliT* coding sequence was also transformed into the genetically distant yeast, *S. cerevisiae* BY4741, under control of the constitutive *SSA2* promoter [24] in plasmid pC210. As can be seen in Figure 6D, yeast transformed with plasmid-encoded *gliT* were capable of growth in the presence of gliotoxin (16 and 64 µg/ml, respectively) depending on whether minimal or rich media was used to support growth, while those transformed with empty vector were unable to grow, irrespective of what media conditions were used. These observations further confirm the pivotal role of *gliT* in mediating resistance to gliotoxin, even in fungal species which do not normally contain the gene or biosynthesise gliotoxin.

## Discussion

Studies into the biosynthesis and pathogenicity of gliotoxin have attracted significant recent attention, stimulated in part by the plethora of fungal genome data now emerging [3], [22]. Here, we demonstrate for the first time that disruption of *gliT*, found within the gliotoxin biosynthetic cluster, but subject to differential regulation, completely sensitizes *A. fumigatus* to exogenous gliotoxin, and abolishes gliotoxin secretion. The possibility that genes adjacent to *gliT* in the gliotoxin gene cluster (*gliF* or *gliH*) play a role in auto-protection is excluded. Thus, we have elucidated a key cellular protective mechanism against the hitherto unknown, potent auto-toxicity of gliotoxin in *A. fumigatus*. Exposure of *A. fumigatus* Δ*gliT* to gliotoxin appears to result in depletion of intracellular GSH since the inhibitory phenotype can be completely relieved by GSH supplementation. Furthermore, we demonstrate the enzymatic functionality of GliT as a gliotoxin reductase and that GliT reactivity is evident in human sera. We demonstrate that *gliT* confers resistance to exogenous gliotoxin, independently of the extent of *gliT* expression, following transformation in naïve hosts, *A. nidulans* and *S. cerevisiae*. Finally, identification of *gliT* complementation in *A. fumigatus* Δ*gliT* ^46645^ and ^26933^, respectively, was selected for in the presence of gliotoxin which supports a selection marker role for *gliT* in *A. fumigatus* transformation experimentation.

To date, the potential requirements for self-protection against gliotoxin, in *A. fumigatus*, have not been studied. The ETP toxin, sirodesmin, is produced by the fungus *Leptosphaeria maculans* with biosynthesis encoded by a multigene cluster similar to that responsible for gliotoxin production in *A. fumigatus* [13]. Deletion of the sirodesmin transporter gene, *sirA*, in *L. maculans* led to increased sensitivity to exogenous sirodesmin and gliotoxin, however the *A. fumigatus* gliotoxin transporter, GliA, was shown to confer resistance to exogenous gliotoxin (10 µM), but not sirodesmin, in *L. maculans* Δ*sirA*. Interestingly, production and secretion of sirodesmin was actually increased by 39% in *L. maculans* Δ*sirA* compared to wild-type and resulted in speculation as to the presence of alternative toxin efflux mechanisms [13]. Based on our observations, we hypothesise that in addition to the likely role of *gliA* in gliotoxin efflux in *A. fumigatus*, GliT may play an essential role in the auto-protective strategy against the deleterious effects of the ETP toxin. Moreover, we predict that *gliT* orthologs in other fungi [22] may play similar, if not identical roles.

Our results indicate that absence of GliT may lead to accumulation of intracellular gliotoxin which is reduced, non-enzymatically, by GSH, analogous to the situation in animal cells as demonstrated by Bernardo *et al*. [17]. The concomitant depletion of intracellular GSH levels, allied to the cytotoxicity of reduced gliotoxin, results in strong growth inhibition, possibly mediated by disruption of the cellular redox status and significant protein modification by gliotoxin. This conclusion is strongly supported by the observation that addition of GSH, during exposure of *A. fumigatus* Δ*gliT* to gliotoxin, effectively completely reverses the cytostatic effects of gliotoxin. While we cannot exclude the possibility that added GSH is merely reducing exogenously added gliotoxin and preventing import of the reduced form, it is clear from Figure 1 that addition of NaBH_4_-reduced gliotoxin results in significant growth inhibition of *A. fumigatus* Δ*gliT* (p<0.05). The observed alleviation of this inhibition (by NaBH_4_-reduced gliotoxin), in the presence of added GSH, supports the proposal that intracellular GSH depletion is a consequence of *gliT* disruption, when growth occurs in the presence of exogenous gliotoxin.

Addition of gliotoxin (up to 20 µg/ml) for 24 h resulted in the complete up-regulation of the gene cluster (except *gliP*) in *A. fumigatus* Δ*gliP*, but not in *A. fumigatus* wild-type [9]. We demonstrate that exposure to exogenous gliotoxin for 3 h does induce GliT expression in *A. fumigatus* wild-type at the transcript and protein level, in fact these data also represent the first confirmed identification of a protein encoded by the gliotoxin biosynthetic cluster. The discrepancy, possibly due to 3 versus 24 h experimental windows, nonetheless, indicates differential GliT expression relative to other *gli* genes. Disruption of *gliZ*, the transcriptional regulator of the gliotoxin biosynthetic cluster, has been shown to result in abolition of gliotoxin production and loss of gliotoxin cluster gene expression [14]. Our data demonstrate that although growth of *A. fumigatus* Δ*gliZ* and *gliZ* ^c^ is unaffected by exogenous gliotoxin, *gliZ* expression is up-regulated in response to exogenous gliotoxin exposure in *A. fumigatus* ATCC46645, but to a lesser extent than that of *gliT* (Figure 2). In addition, we have shown that *gliT* expression is induced by gliotoxin addition to liquid cultures of *A. fumigatus* Δ*gliZ* thereby confirming the independent regulation of *gliT* expression to other cluster components (e.g., *gliA* and *gliG*). In combination, these observations further confirm the minimal role played by any other component of the *gli* gene cluster in protection against gliotoxin presence since *gliZ* absence results in complete cluster attenuation [14], except for *gliT*.

**Figure 7:**
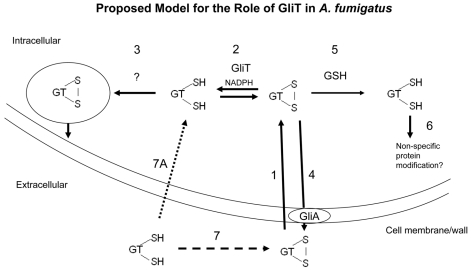
A proposed model for GliT functionality in *A. fumigatus* based on experimental observations. Exogenous gliotoxin enters *A. fumigatus* (**1**) and is converted to the reduced form intracellularly by GliT (gliotoxin reductase activity (**2**)). GliT may also be necessary to oxidize reduced gliotoxin during biosynthesis in *A. fumigatus*. Given the toxicity of the intracellular form of reduced gliotoxin, we predict that it may be imported into intracellular vesicles, possibly with concomitant oxidation for storage (**3**). GliA function to facilitate gliotoxin efflux (**4**) is extrapolated from the observation in *L. maculans* that this protein confers resistance to exogenous gliotoxin [13]. In the absence of GliT, gliotoxin may be alternately reduced by intracellular GSH (**5**) leading to a depletion in GSH and cell death/growth arrest and also modification of other cellular proteins leading to inactivation or activity modification (**6**). In this model, absence of GliT would lead to the build up of gliotoxin within the cell and also the inability to reduce exogenously added gliotoxin. Reduced gliotoxin may not enter but converts to the oxidized form in a time-dependent manner (**7**, **7A**).

A thioredoxin system in *A. nidulans* has recently been described whereby a thioredoxin mutant exhibited decreased growth, impaired reproductive function and altered catalase activity [25]. These authors also identified a thioredoxin reductase (termed AnTrxR) which functions to regenerate reduced thioredoxin in *A. nidulans*. Our BLAST analysis indicates minimal identity between GliT and AnTrxR as well as between GliT and a second putative thioredoxin reductase in *A. fumigatus* (Genbank accession number: EAL85952; 30% identity). This strongly indicates distinct functionality of *gliT* and confirms that alternative thioredoxin reductase activities cannot compensate for loss of *gliT* in *A. fumigatus*. It further appears unlikely that thioredoxin is involved in mediating GliT activity since no thioredoxin reductase present in *A. fumigatus* cell lysates appears capable of compensating for GliT absence. Consequent to its bioinformatic classification as a thioredoxin reductase, GliT has been predicted by many authors to encode disulphide bond formation in gliotoxin and to play a role in gliotoxin biosynthesis [3], [8], [22]. While this ‘gliotoxin oxidase’ activity cannot be ruled out completely, our demonstration that GliT exhibits gliotoxin reductase activity (Figure 5) suggests that *direct* gliotoxin reduction is a pre-requisite for secretion from *A. fumigatus* via a GliT-mediated pathway or as a component of the auto-protective mechanism deployed against exogenous gliotoxin secreted by adjacent fungi in the environment (Figure 7). This hypothesis is firmly supported by the absence of gliotoxin secretion in *A. fumigatus* Δ*gliT* ^26933^. Given the potential of reduced gliotoxin to thiolate cellular proteins, we speculate that reduced gliotoxin may be sequestered into intracellular vesicles where it is converted to the oxidized form, by an unidentified activity, prior to release from the cell by an exocytotic mechanism complementary to GliA-mediated efflux (Figure 7). It remains possible that GliT-mediated gliotoxin oxidase activity may be associated with disulfide bridge closure during gliotoxin biosynthesis when intracellular levels of gliotoxin can be regulated more precisely by the organism. Thus, GliT could be necessary to maintain a balance between reduced and oxidised gliotoxin in *A. fumigatus*. The detection of a molecular ion, with a molecular mass corresponding to a monothiol form of gliotoxin, in culture supernatants from *A. fumigatus* Δ*gliT* is interesting, and we hypothesize that this metabolite may represent a breakdown product of gliotoxin. Future work will involve purification and complete characterization of this molecule. The observation that GliT-specific IgG was present in human sera was unexpected and implies that GliT is either present in inhaled conidia or is expressed during abortive conidial germination in immunocompetent individuals. However, our observation suggests that the option of using normal human sera as a source of immunoaffinity antibodies, following Ig isolation and purification using a recombinant antigen (e.g., GliT), represents a novel approach for readily obtaining monospecific antisera against antigenic *A. fumigatus* proteins.

The animal model system deployed herein appears to distinguish between virulence diminution associated with lack of gliotoxin production, since inoculation with *A. fumigatus* Δ*gliZ* resulted in reduced Gallerial mortality than exposure to wild-type *A. fumigatus*. This result extends previous observations with respect to the potential avirulence of *A. fumigatus* Δ*gliZ* [14]. However, the relatively equivalent virulence observed for *A. fumigatus* wild-type and Δ*gliT*, whereby the latter does not produce gliotoxin is somewhat at variance with the *A. fumigatus* Δ*gliZ* findings. We suggest that alterations in the levels of additional metabolites in *A. fumigatus* Δ*gliZ*, as noted in [14], or a possible cytotoxic role in *G. mellonella* for the putative monothiol form of gliotoxin secreted by *A. fumigatus* Δ*gliT* may account for this dichotomy. Our demonstration that *gliT* is expressed independently of other cluster components implies that previous virulence model experimentation, involving *gliP*- and *gliZ* –deficient mutants [9]–[12], may require interpretation in light of the possibility of independently regulated *gliT* expression, or GliT functionality. Indeed, if it is ever demonstrated that *gliT* expression occurs in the absence of *gli* cluster expression/gliotoxin biosynthesis (as has been demonstrated herein for *A. fumigatus* Δ*gliZ*), or is regulated by factors other than exposure to exogenous gliotoxin, then consideration may need to be given to this phenomenon in future studies. This consideration is based on the fact that independent regulation of *gliT* may have enabled acquisition of functionality beyond a role in gliotoxin biosynthesis or auto-protection.

Genetic modification of filamentous fungi for the improved production of food additives, industrial enzymes or pharmaceuticals is an ongoing requirement of the biotechnological industry [26], [27]. Antibiotic-producing fungi are continually subjected to strain improvement, with a concomitant requirement for new selection markers, to increase product yield and decrease the level of unwanted side-products [28]. Our observation that *gliT* complementation in *A. fumigatus* can be selected for in the presence of gliotoxin, without the use of conventional selection markers, and that transformation of *A. nidulans* and *S. cerevisiae* with *gliT* confers enhanced resistance to gliotoxin offers the possibility of using the *gliT*/gliotoxin combination to select for fungal transformation. Moreover, acquired gliotoxin resistance in *A. nidulans* and *S. cerevisiae* resulting from *gliT* presence, underpins the important role played by this gene in mediating resistance to exogenous gliotoxin. Gliotoxin isolated from cultures of a marine fungus from the genus *Pseudallescheria* has been shown to possess both anti-bacterial and free-radical scavenging capability whereby an MIC_50_ of 1 µg/ml was observed against methicillin-resistant *Staphylococcus aureus* [29]. Gliotoxin may also provide a competitive advantage for *A. fumigatus* when grown in the presence of other fungi [30]. In this regard, gliotoxin production has been detected when *A. fumigatus* was co-cultured, at both 30 and 37°C, with a range of other *Aspergillus spp*., leading the authors to speculate that co-expression of resistance genes may allow toxin producers to resist the effects of their own biological arsenal in competitive co-culture situations [30]. The parallel between this supposition, and our observation of GliT-mediated resistance to exogenous gliotoxin, is vivid.

The vast majority of literature surrounding the role of gliotoxin in *A. fumigatus* focuses on its function as a cytotoxic molecule which has deleterious effects on cells within infected individuals and exhibits anti-microbial activity [5], [6], [9]–[12], [29], [30]. However, based on our observations and significant other literature [16], [18], [31], a credible alternative hypothesis is that gliotoxin may actually be part of the intracellular antioxidant defense system within *A. fumigatus*, and is a molecule, analogous to thioredoxin or 2-cys peroxiredoxin, which may undergo rapid changes in redox status to buffer against specific exogenous or endogenous oxidants. In other words, the cytotoxic effects of gliotoxin in infected host cells may actually be an indirect consequence of its role within *A. fumigatus*. This alternative hypothesis is not without support. Firstly, Watanabe *et al.* [31] have shown that the cytotoxicity of *A. fumigatus* culture filtrates was significantly attenuated, or absent, when cultures were grown under reduced aerobic or anaerobic conditions. Interestingly, gliotoxin production was detectable by GC-MS analysis from aerobic but not in reduced aerobic culture supernatants. Although Watanabe *et al*. concluded that their results indicated that gliotoxin production is increased to facilitate fungal pathogenicity (mimicking the aerobic lung environment), an alternative conclusion, which is in accordance with our thinking, is that gliotoxin production is actually elevated to cope with increased oxygen levels and that secretion of gliotoxin forms part of the gliotoxin homeostasis control mechanism within *A. fumigatus* to prevent the side-effect of intracellular oxidative stress. As noted earlier, in animal cells it has been shown that gliotoxin may substitute for 2-cys peroxiredoxin activity in HeLa cells by accepting electrons from NADPH via the thioredoxin reductase–thioredoxin redox system to reduce H_2_O_2_ to H_2_O. In this way, nanomolar levels of gliotoxin may actually protect against intracellular oxidative stress [18]. Additionally, as demonstrated by Srinivasan *et al*. [16], oxidized gliotoxin facilitates selective protein inactivation in the presence of molecular oxygen which, we hypothesise, could prevent global intracellular damage due to resultant reactive oxygen species. Moreover, a protective role for gliotoxin against environmental stress in *A. fumigatus* has been considered [2], [13]. Our observations and consequent hypothesis now provide a vehicle to explore this proposal.

In summary, we have demonstrated that GliT plays a major auto-protective role against gliotoxin toxicity in *A. fumigatus* which points to alternative gliotoxin functionality in *A. fumigatus*. From a utilitarian viewpoint, *gliT*/gliotoxin sensitivity represents a potential new selection marker strategy for fungal transformation. The trans-fungal implications of our observations remain to be explored.

## Materials and Methods

### Ethics statement

This study was conducted according to the principles expressed in the Declaration of Helsinki. Ethical permission was obtained from The Ethics Committee of NUI Maynooth for the use of human serum specimens. Anonymous serum specimens were obtained with the signed agreement of the Irish Blood Transfusion Service.

### Strains, growth conditions, and general DNA manipulation

In general, *A. fumigatus* strains (Table 1) were grown at 37°C in *Aspergillus* minimal media (AMM). AMM contained 1% (w/v) glucose as carbon-source, 5 mM ammonium tartarate as nitrogen-source, and trace elements according to Pontecorvo *et al*. [32]. Liquid cultures were performed with 200 ml AMM in 500 ml Erlenmeyer flasks inoculated with 10^8^ conidia. For growth assays, 10^4^ conidia of the respective strains were point inoculated on AMM plates, containing the relevant supplements and incubated for 48 h at 37°C.

**Table 1:** *A. fumigatus* and *A. nidulans* strains used in this study.

Strain	Genotype	Reference
ATCC46645	Wild-type	Hearn *et al.* [43]
ATCC26933	Wild-type	Taylor *et al.* [44]
Δ*gliZ*	Δ*gliZ*::*pyrG*	Bok *et al.* [14]
*gliZ* ^c^	Δ*gliZ*::*gliZ::hygB*	Bok *et al.* [14]
Δ*gliT^46645^*	ATCC46645; *gliT*::*ptrA*	This study
Δ*gliT^26933^*	ATCC26933; *gliT::ptrA*	This study
*gliT* ^C^	Δ*gliT*; Δ*gliT*::*gliT*	This study
*gliT^gfp^*	Δ*gliT^46645^*; (p)*gliTgfp*	This study
*A. nidulans WGTRAN*	Wild-type	Oberegger *et al.* [45]
*An^gliT^*	TRAN; (p)*gliT*	This study
Δ*gliH*	ATCC26933; *gliH::ptrA*	This study
Δ*gliT^26933gliH^*	ATCC26933; *gliT::ptrA;*	This study
	(p)gliH; (p)AN7-1	
*gliH^C^*	Δ*gliH*; (p)*gliH*; (p)AN7-1	This study

TOPO TA cloning system (Invitrogen) and TOP10 *E. coli* cells (F-*mcrA* Δ(*mrr-hsd*RMS-*mcr*BC) φ80*lac*ZΔM15 Δ*lac*X74 *rec*A1 *ara*D139 *gal*U *gal*K Δ (*ara-leu*)7697 *rps*L (Str^R^) *end*A1 *nup*G) were used for general plasmid DNA propagation and *A. fumigatus* genomic DNA was purified using a ZR Fungal/Bacterial DNA Kit (Zymoresearch).

### Generation of *A. fumigatus* mutant strains

For generating Δ*gliT* mutant strains, the bipartite marker technique was used [20]. Briefly, *A. fumigatus* strains ATCC46645 and ATCC26933 were co-transformed with two DNA constructs, each containing an incomplete fragment of a pyrithiamine resistance gene (*ptrA*) [21] fused to 1.2 kb, and 1.3 kb of *gliT* flanking sequences, respectively. These marker fragments shared a 557 bp overlap within the *ptrA* cassette, which served as a potential recombination site during transformation. During transformation, homologous integration of each fragment into the genome flanking *gliT* allows recombination of the *ptrA* fragments and generation of the intact resistance gene at the site of recombination. Two rounds of PCR generated each fragment. First, each flanking region was amplified from ATCC46645 genomic DNA using primer ogliT1 and ogliT4 for flanking region A (1.3 kb), and ogliT-2 and ogliT-3 for flanking region B (1.2 kb). Subsequent to gel-purification, the fragments were digested with *Spe*I and *Hind*III, respectively. The *ptrA* selection marker was released from plasmid pSK275 (a kind gift from Sven Krappmann, Goettingen, Germany) by digestion with *Spe*I and *Hind*III, and ligated with the two flanking regions A and B described above. For generation of Δ*gliT,* two overlapping fragments were amplified from the ligation products using primers ogliT-5 and optrA-2 for fragment C (2.6 kb) and primers ogliT-6 and optrA-1 for fragment D (2.2 kb). Subsequently ATCC46645 and ATCC26933 were transformed simultaneously with the overlapping fragments C and D. In the generated mutant allele of Δ*gliT-ptrA* the deleted region comprises amino acids 1–325 of *gliT*.

For reconstitution of the Δ*gliT* strain with a functional *gliT* copy, a 3.2 kb PCR fragment, amplified using primers ogliT-5 and ogliT-6, was subcloned into pCR2.1-TOPO (Invitrogen). The resulting 7.1 kb p*gliT* was linearised with *Aat*II and used to transform *A. fumigatus* Δ*gliT* protoplasts. Taking advantage of the decreased resistance of the Δ*gliT* mutant to exogenous added gliotoxin Δ*gliT* protoplasts were transformed with p*gliT* and screened for wild-type resistance to gliotoxin for genetic complementation. Positive deletion- and reconstituted- strains were screened by Southern analysis (Figure S1) and DIG-hybridisation probes were generated using primers ogliT-5 and ogliT-4.

To obtain knock-out constructs for the deletion of *gliH* a 5′ flanking region with oligos ogliH1 and ogliH4 was amplified. For the 3′ flanking region a PCR with oligos ogliH2 and ogliH3 was performed. Amplicons were digested with *Spe*I and *Hind*III, respectively. Resulting fragments were ligated to a *ptrA* cassette, released from pSK275 via *Spe*I and *Hind*III digest. Final PCRs were obtained using oligos ogliH5/optrA2 and ogliH6/optrA1 and used for transformation.

To complement Δ*gliH* and Δ*gliT^26933^* with a functional copy of *gliH*, oligos ogliT7 and M13 were used to amplify a PCR-fragment using pgliT as template. This fragment digested with *EcoR*I and *Sac*II was cloned into pBS-KS (Stratagene), resulting in pgliH. Together with pAN7-1 [33], pgliH was used to complement *A. fumigatus* Δ*gliH* and Δ*gliT^26933^*.

GliT was C-terminally fused in frame to gfp (green fluorescent protein) to determine its subcellular localisation. To this end, a fragment containing *gliT* was amplified using oligos ogliT-5-*Sph*I and ogliT-16. The resulting 2.2 kb fragment was sub-cloned into pCR2.1-TOPO (Invitrogen) and sequenced. Via *Sph*I digest a fragment containing the *gliT* promoter region and the coding sequence was released and cloned into the corresponding *Sph*I site of pgfp, resulting in pgliTgfp. To obtain pgfp, a *gfp* containing fragment was released from pUCG-H [34] via *Sma*I and *Sac*I and subcloned into the corresponding *Eco*RV and *Sac*I sites of pGEM5zf+ (Promega). The plasmid pgliTgfp was used to transform Δ*gliT* protoplasts via co-transformation using a phleomycin resistance gene. Phleomycin resistant transformants carrying an in-frame *gliT-gfp* fusion were used to localize GliT using fluorescence microscopy. Positive, GliT-GFP harbouring strains were screened by Southern analysis and hybridization probes were generated using oligos ogliT-7 and ogliT-8. *A. fumigatus* transformation was carried out according to Tilburn *et al*. [35]. In order to obtain homokaryotic transformants, colonies from single homokaryotic spores were picked and single genomic integration was confirmed by PCR (data not shown) and Southern blot analysis.

### Northern analysis

RNA was isolated using TRI-Reagent (Sigma-Aldrich). Equal concentrations of total RNA (10 µg) were size-separated on 1.2% agarose-2.2 M formaldehyde gels and blotted onto Hybond N+ membranes (Amersham Biosciences). The hybridisation probes used in this study were generated by PCR using primers ogliA1 and ogliA2 for AFUA_6G09710, ogliG7 and ogliG8 for AFUA_6G09690, ogliT7 and ogliT8 for AFUA_6G09740, and ogliZ1 and ogliZ2 for AFUA_6G09630. All primers used in this study are listed in Table 2.

**Table 2:** Primers used in this study.

Primer	Sequence (5′–3′)
ogliA-1	TGG ATC GTT GAT CTG CGC
ogliA-2	ATG GCC TGG TAT CCG ATC
ogliG-7	GAC CCT CCG ATC TTG TAG
ogliG-8	TTC TCG CCA TGG CCA AAC
ogliT-1	AGC GCA TTG GAC AGG TTG TAG
ogliT-2	GGA CAC GTC TAG CAT GGA CTG G
ogliT-3	GCT AAG CTT TTG CCG GAG TTT CGT CTC
ogliT-4	GGA CTA GTT ATG CGC GAG AGT AGT GG
ogliT-5	TCT GCG CTT CTT GAT CGG
ogliT-6	ACG GTG CTG GGA ATG ATC
ogliT-7	GTC GAC GTG CTC ATC ATC
ogliT-8	GCC AAA GAT CCC ATC GAC
ogliT-5-*Sph*I	CGG CAT GCT CTG CGC TTC TTG ATC GG
ogliT-16	AAA GCA TGC TAG CTCCTG ATC GAG ACG
ogliZ-1	GCT ATG CAG GAT GTG TCG
ogliZ-2	CGG CCA TGC TAA TAC TGC
optrA1	GAG GAC CTG GAC AAG TAC
optrA2	CAT CGT GAC CAG TGG TAC
ogliT-*Bgl*II	CCA GAT CTA TGT CGA TCG GCA AAC TAC
ogliT-*Not*I	ATA GCG GCC GCC TAT AGC TCC TGA TCG AGA
ogliH1	CAT GCA CAA CGT CCT CGG ATG
ogliH2	GCT CCT GGG GAT TCT GAG CGC
ogliH3	AAC AAG CTT AGA ATG GGC AGT TGG ACG
ogliH4	GCT ACT AGT GAA GAT CTG TCT GCC GTC
ogliH5	TCC ACC ATC CAG TTC CAG
ogliH6	GCG GTG CAG TGA ACT AAC
M13	GTAAAACGACGGCCAGT
M13rev	AACAGCTATGACCATG
Sc-gliT-F	CCCGGGCATATG TCGATCGGCAAACTACTCTCAAC
Sc-gliT-R	CCCGGGGCATGC CTATAGCTCCTGATCGAGACGAAAC

Added restriction enzyme sites are underlined.

### Proteomic analysis of GliT expression

*A. fumigatus* ATCC26933 was cultured (*n* = 3) for 21 h in Sabouraud media followed by gliotoxin addition for 3 h (final concentration: 14 µg/ml). Control cultures (*n* = 3), where gliotoxin was not added, were also performed. Mycelia were harvested, lysed and subject to MALDI-ToF mass spectrometric analysis as previously described [36] and Imagemaster analysis (GE Healthcare).

### Analysis of gliotoxin production

To analyze gliotoxin, or related metabolite production, *A. fumigatus* wild-type and mutant strains were grown up at 37°C for 72 h in Czapeks-Dox. Supernatants were chloroform extracted overnight and fractions were lyophilized to complete dryness. Samples were resolubilised in MeOH and analysed using a reversed phase HPLC as described in [37] and LC-MS (Agilent 6340 ETD LC-MS system). Samples (1 µl) were loaded onto a Zorbax 300SB C-18 Nano-HPLC Chip (150 mm×75 µm, Agilent) with 0.1%(v/v) formic acid (0.6 µl/min), and compounds eluted by an increasing 0.1%(v/v) formic acid, acetonitrile gradient (90%(v/v) final). Eluted compounds were directly ionised and analysed by ion trap mass spectrometer (Agilent). For each round of MS the two most abundant compounds were automatically selected for MS^n^ analysis. Gliotoxin was identified by its whole mass of 326.4 m/z and its characteristic MS^n^ fragmentation pattern (263, 245 and 227 m/z). LC-ToF analysis was performed using an Agilent HPLC 1200 series using electrospray ionisation inputted into a ToF (Agilent). LC separation was via an XDB C_18_ column (4.6×150 mm) using a water/acetonitrile (both containing 0.1% (v/v) formic acid) gradient at a flow rate of 0.5 ml/min. The gradient was started at 50% (v/v) acetonitrile, which was increased to 100% acetonitrile in 10 min; 100% acetonitrile was maintained for 5 min before the gradient was returned to starting conditions. Spectra were collected at 0.99 spectra per second.

### Cloning and expression of *gliT*

The *gliT* sequence was amplified from cDNA using primers incorporating terminal *Xho*I *and Hind*III sites to facilitate downstream cloning. PCR products were cloned into the pCR2.1 cloning vector (Invitrogen, Carlsbad, CA, USA) according to the manufacturer's instructions. *gliT* was subsequently cloned into the pProEX-Htb expression vector (Invitrogen). Ligations were performed using Quickstick ligase (Bioline, London, UK) according to the manufacturer's instructions. pPXA*gliT*, the resultant expression vector was transformed into *E. coli* strain BL21 by standard protocols. Expression of GliT was induced by the addition of isopropyl β-D-thiogalactoside (IPTG; to 0.6 mM) and monitored by SDS-PAGE and Western blot analysis. Recombinant GliT purification was undertaken by differential extraction. Protein concentrations were determined using the Bradford method [38] with bovine serum albumin as a standard.

### Purification of native GliT from *A. fumigatus* by ion-exchange chromatography

*A. fumigatus* ATCC46645 mycelia were ground in liquid nitrogen and lysed in ice-cold lysis buffer as described [36] following incubation with gliotoxin (10 µg/ml) for 3 h). Following centrifugation (12,000 *g*; 30 min), the lysate supernatant (176 ml) was ammonium sulphate precipitated (10, 20, 50 and 70% ammonium sulphate). The 50% pellet was resuspended in 20 mM Bis-Tris propane pH 7.6 and dialysed three times against 50 volumes of the same buffer at 4°C. The dialysate was centrifuged (12,000 *g*; 20 min) and filtered (0.45 µm) to remove particulates. The dialysate was loaded onto an equilibrated Q-Sepharose ion-exchange (IEX) column (4 ml) at a flow rate of 1 ml/min. The column was washed with 20 mM Bis-Tris propane pH 7.6 before bound protein was eluted using an NaCl gradient (0.5 M final). Absorbance detection was at 280 nm and 454 nm. Collected fractions were subjected to SDS-PAGE, Western blot and activity analysis for GliT.

### Immunoaffinity purification of human IgG [anti-GliT]

Serum specimens (provided by the Irish Blood Transfusion Service, Dublin, Ireland according to institutional guidelines) containing high titer IgG [anti-GliT] were pooled, diluted 1 in 4 in PBS, and applied to a GliT-Sepharose affinity column (0.5 ml), prepared as per manufacturer's instructions. After removal of unbound proteins by PBS washing, immobilised IgG [anti-GliT] was eluted using 100 mM glycine pH 2.8, followed by immediate neutralization using 100 mM Trizma base pH 8.3. Resultant immunoaffinity purified (IAP) IgG [anti-GliT] was used to detect native GliT by Western analysis.

### GliT activity assay and removal of native GliT from *A. fumigatus* by IAP pulldown

*A. fumigatus* ATCC46645 mycelia were ground in liquid nitrogen and lysed in ice-cold lysis buffer and bead-beating as described elsewhere [36]. Following centrifugation (12,000 *g*; 30 min), the lysate supernatants were used to determine gliotoxin reductase activity (ΔA_340 nm_) in the presence of gliotoxin (9 µM) and NADPH (200 µM) at pH 7.2 (a modified version of Hill *et al*. [39]). *A. fumigatus* cell lysates were also subjected to ion-exchange chromatography and a pooled IEX fractions (250 µl) incubated with IAP human IgG [anti-GliT] (100 µl) followed by Protein A-Sepharose addition and centrifugation (10,000 *g*; 10 min). Supernatant activity analysis as described above.

### GliT-GFP confocal microscopy

*A. fumigatus gliT^gfp^* and ATCC46645 mycelia were grown in cell culture six well plates (Corning Inc.) for 21 h before induction with (or without) gliotoxin (5 µg/ml). Mycelia were removed from the wells and centrifuged (12,000 *g*; 5 min). Supernatants were stored while pellets were resuspended in DAPI staining solution and incubated (5 min) at room temperature. The stained mycelia were centrifuged and washed with deionised H_2_O before resuspension in the original supernatant. Aliquots of these preparations were analysed for GliT-GFP presence and DAPI fluorescence on an Olympus Fluoview 1000 confocal microscope.

### Virulence model

*G. mellonella* larvae (*n* = 10) were inoculated into the hind pro-leg with 10^6^ *A. fumigatus* conidia in 20 µl (per larva) [37]. In addition, one cohort of larvae was pre-treated with gliotoxin (0.5 µg/larva in 20 µl). Control treatments were included to ensure that neither the injection procedure, or the incubation period, were responsible for any mortality observed. These controls involved *G. mellonella* larvae injected with 20 µl of sterile PBS or gliotoxin alone. *G. mellonella* larvae were placed in Petri-dishes and incubated in the dark at 30°C. Mortality rates were recorded for 72 h post-injection. Mortality was assessed based on lack of movement in response to stimulation and discolouration (melanisation) of the cuticle.

### Generation of *gliT*-encoding *Aspergillus nidulans* and *Saccharomyces cerevisiae*

To introduce *gliT* in *A. nidulans* TRAN, a plasmid containing *gliT* coding sequence under the control of a constitutive *otef* [40] promoter was used. Therefore, a 1.1 kb fragment containing *gliT* was amplified using ogliT-*Bgl*II and ogliT-*Not*I and subcloned into pCR2.1-TOPO (Invitrogen). A 0.9 kb fragment containing an *otef* promoter was released via *Bam*HI/*Kpn*I digest from plasmid pUCG-H [34] and cloned into the respective sites into pGliT-*Bgl*II-*Not*I. Transformation was performed as described for *A. fumigatus*.

The *S. cerevisiae* strain used in this study was BY4741 (*MATa his3D1 leu2D0 met15D0 ura3D0*) and was purchased from Euroscarf. Rich and minimal yeast medium was as described in [41], and gliotoxin was added to the desired concentration to cooled molten agar. To monitor the effects of GliT expression in *S. cerevisiae gliT* was amplified from *A. fumigatus* (ATCC46645) using PCR with primers Sc-gliT-F and Sc-gliT-R (Table 2), and cloned into the yeast shuttle vector pC210 [42]. Plasmids pC210 harbors the *SSA1* coding sequence under control of the constitutive *SSA2* promoter. Following digestion of pC210 with *Nde*I and *Sph*I to remove the *SSA1* coding sequence, similarly digested *gliT* PCR product was ligated into pC210 to create pC-GliT. Thus, pC-GliT harbors *A. fumigatus gliT* under control of the strong constitutive *S. cerevisiae SSA2* promoter. The integrity of pC-GliT was confirmed by sequencing.

To test the sensitivity of yeast to gliotoxin, BY4741 harboring either vector alone (pRS315) or pC-GliT was grown to mid-exponential phase (3×10^6^ cells/ml). Cells were harvested and resuspended in rich medium to a concentration of 5×10^6^ cells/ml. Cells were serially diluted and were plated onto rich or minimal medium containing the desired concentration of gliotoxin, using a multi-pronged replicator. Plates were incubated at 30°C for 48 h with further monitoring of plates at room temperature for 72 h.

### Accession numbers

The proteins named herein are available at Genbank under the following Accession numbers: GliA (AAW03302); GliF (AAW03300); GliG (AAW03304); GliH/AFUA_6G09745 (EAL88826); GliT (AAW03299) and GliZ (AAW03310).

## Supporting Information

Protocol S1 Supplementary data.
(0.03 MB DOC)

Figure S1 (A) Deletion of *gliT* and *gliH* in *A. fumigatus* ATCC46645 and 26933, respectively. Southern analysis of Δ*gliT* mutant versus wild-type DNA for *A. fumigatus* ATCC46645 and ATCC26933, respectively. Here, a DIG-labelled probe was used to detect the predicted presence of 3.3 and 6.4 kb fragments in *Xba*I restricted Δ*gliT* and wild-type DNA, respectively. (B) Southern Blot analysis of Δ*gliT* complemented strains (*gliT^C^*). Genomic DNA of wild-type and complemented strains was digested with *Nar*I (ATCC46645) and *Apa*I (ATCC26933), respectively and probed using a DIG-labelled probe amplified using oligos ogliT-4 and ogliT-5. (C) Southern Blot analysis of Δ*gliH*, Δ*gliH*-complemented strains (*gliH^C^*) and Δ*gliT*-complemented with gliH (Δ*gliT^26933gliH^*). Genomic DNA of wild-type and respective mutant strains was digested with *Nde*I (ATCC26933) and probed using a DIG-labelled probe amplified using oligos ogliH-4 and ogliH-5. (1) *A. fumigatus* ATCC26993, (2) Δ*gliH*, (3) *gliH^C^*, (4) Δ*gliT*, (5) Δ*gliT^26933gliH^*.
(1.30 MB DOC)

Figure S2 Phenotypic analysis of *A. fumigatus* ATCC46645 (wild-type) and Δ*gliT* strains in the presence of gliotoxin (GT). Compared to wild-type, gliotoxin (5 µg/ml) significantly inhibits Δ*gliT* growth in minimal medium (MM) and completely inhibits Δ*gliT* growth in both MM and Sabouraud medium (10 µg/ml).
(0.04 MB DOC)

Figure S3 Peptide mass spectrum of GliT from *A. fumigatus* ATCC26933, a component of the gliotoxin biosynthetic cluster (33% sequence coverage). This MALDI-ToF identification represents the first proteomic confirmation of the expression of a component of the gliotoxin biosynthetic cluster.
(0.11 MB DOC)

Figure S4 Analysis of gliotoxin, and related metabolite, production in *A. fumigatus* mutant strains. (A) Gliotoxin was detectable by RP-HPLC (data not shown) and LC-MS in *A. fumigatus* ATCC26933 *gliT* ^c^ with identical molecular mass and fragmentation pattern to commercially available gliotoxin and as reported in [10]. (B) LC-ToF analysis of RP-HPLC purified gliotoxin-related metabolite (Figure 3B) from *Aspergillus fumigatus* Δ*gliT* ^26933^. MS spectrum shows the presence of a high abundance molecular ion (Retention time  = 9.153 min) with m/z 279.0796 (M+H)^+^ (557.1497 (2M+H)^+^) which corresponds precisely to a predicted molecular formula of C13 H14 N2 O3 S - a putative monothiol form of gliotoxin. (C) LC-MS analysis analysis of RP-HPLC purified gliotoxin-related metabolite (Figure 3B) from *Aspergillus fumigatus* Δ*gliT* ^26933^. Using a manual approach, LC-MS software identified five molecular species with m/z 279.0. The most intense peaks (1 and 5) were subjected to MS2 analysis and both yielded identical fragments ions of m/z 261.1, 231.0 and 203.1. Notably, peak 1 eluted from LC-MS and LC-ToF with an identical retention time (9.1 min) (D) Gliotoxin production was undetectable in *A. fumigatus* Δ*gliH^26933^*, by RP-HPLC and LC-MS (data not shown), thereby indicating a role for this gene in either gliotoxin biosynthesis or secretion.
(0.43 MB DOC)

Figure S5 Recombinant GliT expression. (A) SDS-PAGE and (B) Western blot analysis of recombinant GliT expression and solubility. Lane 1 contains non-transformed BL21 (DE3) cells. Lane 2 contains non-induced cell extract and lanes 3–5 contain induced cell extracts taken 1–3 h post-induction with 0.6 mM IPTG. Lane 6 and 7 contain soluble and insoluble cell extracts respectively. Lane 8 contains His-tag positive control and lane 9 contains non-reducing cell extract- monomeric (m) and dimeric (d) forms of GliT are evident. Lane M contains molecular mass marker.
(2.29 MB DOC)

Figure S6 Confirmation of recombinant GliT identity by MALDI-ToF mass spectrometry (21% sequence coverage).
(0.03 MB DOC)

Figure S7 (A) Partial purification and immunological identification of GliT. Absorbance (A280 nm and A454 nm) versus elution volume (ml) for a Q-Sepharose ion-exchange fractionation of GliT dialysate (post-ammonium sulphate precipitation). (B) SDS-PAGE analysis of Q-Sepharose ion-exchange chromatography (IEX) fractions. (C) Western blot analysis of Q-Sepharose IEX fractions using human IgG[anti-GliT] and anti-human IgG-HRP conjugate with ECL detection. These fractions were pooled and used for activity and immunological analysis as shown in Figures 4 and 5.
(0.29 MB DOC)

Figure S8 Gliotoxin induces expression of GliT-GFP expression in *A. fumigatus*. (A) Basal expression of GliT-GFP expression, determined by confocal fluorescence microscopy in *A. fumigatus* in the absence of added gliotoxin. Panel I: GliT-GFP fluorescence, panel II: DAPI nuclear staining and panel III: Image merge. (B) Enhanced expression of GliT-GFP throughout mycelia following exposure to gliotoxin (5 µg/ml). Panel I: GliT-GFP fluorescence, panel II: DAPI nuclear staining and panel III: image merge. (C) Fluorescence intensity for DAPI (blue) and GFP (green) are shown. Yellow line corresponds to the fluorescence intensities depicted with a red arrow. Intensities demonstrate localisation of GliT-GFP in cytoplasm and nuclei.
(1.42 MB DOC)

Figure S9 Expression of GliT-GFP restores resistance to exogenous gliotoxin. Phenotypes of *A. fumigatus* ATCC46645 (WT), Δ*gliT* ^46645^, *gliT^C^* and *gliT^gfp^*. Conidia of the respective strain were point inoculated on AMM plates in the absence and presence of gliotoxin (5 and 10 µg/ml. respectively) and incubated for 40 h at 37°C.
(0.62 MB DOC)

Figure S10 Virulence assay of *A. fumigatus* wild-type, Δ*gliZ*, Δ*gliT* and *gliT^c^*. (A and B) *G. mellonella* challenged with *A. fumigatus* Δ*gliZ* [14], corresponding wild-type, Δ*gliT* ^26933^ and *gliT* ^c^ in the presence (A) (6 ng, pre-incubation 2 hr prior to conidial challenge) and absence (B) of gliotoxin.
(0.24 MB DOC)

## References

[1] Gardiner DM, Waring P, Howlett BJ (2005). The epipolythiodioxopiperazine (ETP) class of fungal toxins: distribution, mode of action, functions and biosynthesis.. Microbiology.

[2] Kwon-Chung KJ, Sugui JA (2008). What do we know about the role of gliotoxin in the pathobiology of *Aspergillus fumigatus*?. Med Mycol.

[3] Fox EM, Howlett BJ (2008). Biosynthetic gene clusters for epipolythiodioxopiperazines in filamentous fungi.. Mycol Res.

[4] Hurne AM, Chai CL, Waring P (2000). Inactivation of rabbit muscle creatine kinase by reversible formation of an internal disulfide bond induced by the fungal toxin gliotoxin.. J Biol Chem.

[5] Tsunawaki S, Yoshida LS, Nishida S, Kobayashi T, Shimoyama T (2004). Fungal metabolite gliotoxin inhibits assembly of the human respiratory burst NADPH oxidase.. Infect Immun.

[6] Nishida S, Yoshida LS, Shimoyama T, Nunoi H, Kobayashi T (2005). Fungal metabolite gliotoxin targets flavocytochrome b558 in the activation of the human neutrophil NADPH oxidase.. Infect Immun.

[7] Gardiner DM, Cozijnsen AJ, Wilson LM, Pedras MS, Howlett BJ (2004). The sirodesmin biosynthetic gene cluster of the plant pathogenic fungus *Leptosphaeria maculans*.. *Mol Microbiol*.

[8] Gardiner DM, Howlett BJ (2005). Bioinformatic and expression analysis of the putative gliotoxin biosynthetic gene cluster of *Aspergillus fumigatus.*. FEMS Microbiol Lett.

[9] Cramer RA, Gamcsik MP, Brooking RM, Najvar LK, Kirkpatrick WR (2006). Disruption of a nonribosomal peptide synthetase in *Aspergillus fumigatus* eliminates gliotoxin production.. Eukaryot Cell.

[10] Kupfahl C, Heinekamp T, Geginat G, Ruppert T, Härtl A (2006). Deletion of the *gliP* gene of *Aspergillus fumigatus* results in loss of gliotoxin production but has no effect on virulence of the fungus in a low-dose mouse infection model.. Mol Microbiol.

[11] Sugui JA, Pardo J, Chang YC, Zarember KA, Nardone G (2007). Gliotoxin is a virulence factor of *Aspergillus fumigatus*: *gliP* deletion attenuates virulence in mice immunosuppressed with hydrocortisone.. Eukaryot Cell.

[12] Spikes S, Xu R, Nguyen CK, Chamilos G, Kontoyiannis DP (2008). Gliotoxin production in *Aspergillus fumigatus* contributes to host-specific differences in virulence.. J Infect Dis.

[13] Gardiner DM, Jarvis RS, Howlett BJ (2005). The ABC transporter gene in the sirodesmin biosynthetic gene cluster of *Leptosphaeria maculans* is not essential for sirodesmin production but facilitates self-protection.. Fungal Genet Biol.

[14] Bok JW, Chung D, Balajee SA, Marr KA, Andes D (2006). GliZ, a transcriptional regulator of gliotoxin biosynthesis, contributes to *Aspergillus fumigatus* virulence.. Infect Immun.

[15] Balibar CJ, Walsh CT (2006). GliP, a multimodular nonribosomal peptide synthetase in *Aspergillus fumigatus*, makes the diketopiperazine scaffold of gliotoxin.. Biochemistry.

[16] Srinivasan U, Bala A, Jao SC, Starke DW, Jordan TW (2006). Selective inactivation of glutaredoxin by sporidesmin and other epidithiopiperazinediones.. Biochemistry.

[17] Bernardo PH, Brasch N, Chai CL, Waring P (2003). A novel redox mechanism for the glutathione-dependent reversible uptake of a fungal toxin in cells.. J Biol Chem.

[18] Choi HS, Shim JS, Kim JA, Kang SW, Kwon HJ (2007). Discovery of gliotoxin as a new small molecule targeting thioredoxin redox system.. Biochem Biophys Res Commun.

[19] Chamilos G, Lewis RE, Lamaris GA, Albert ND, Kontoyiannis DP (2008). Genomewide screening for genes associated with gliotoxin resistance and sensitivity in *Saccharomyces cerevisiae.*. Antimicrob Agents Chemother.

[20] Nielsen ML, Albertsen L, Lettier G, Nielsen JB, Mortensen UH (2006). Efficient PCR-based gene targeting with a recyclable marker for *Aspergillus nidulans*. Fungal Genet Biol.

[21] Kubodera T, Yamashita N, Nishimura A (2000). Pyrithiamine resistance gene (*ptrA*) of *Aspergillus oryzae*: cloning, characterization and application as a dominant selectable marker for transformation.. *Biosci Biotechnol Biochem*.

[22] Patron NJ, Waller RF, Cozijnsen AJ, Straney DC, Gardiner DM (2007). Origin and distribution of epipolythiodioxopiperazine (ETP) gene clusters in filamentous ascomycetes.. BMC Evol Biol.

[23] Galagan JE, Calvo SE, Cuomo C, Ma LJ, Wortman JR (2005). Sequencing of *Aspergillus nidulans* and comparative analysis with *A. fumigatus* and *A. oryzae*.. Nature.

[24] Hjorth-Sørensen B, Hoffmann ER, Lissin NM, Sewell AK, Jakobsen BK (2001). Activation of heat shock transcription factor in yeast is not influenced by the levels of expression of heat shock proteins.. Mol Microbiol.

[25] Thön M, Al-Abdallah Q, Hortschansky P, Brakhage AA (2007). The thioredoxin system of the filamentous fungus *Aspergillus nidulans*: impact on development and oxidative stress response.. J Biol Chem.

[26] LeClerque A, Wan H (2007). Novel dominant selection marker for the transformation of fungi.. US Patent.

[27] Archer DB, Dyer PS (2004). From genomics to post-genomics in *Aspergillus.*. Curr Opin Microbiol.

[28] Rodríguez-Sáiz M, Lembo M, Bertetti L, Muraca R, Velasco J (2004). Strain improvement for cephalosporin production by *Acremonium chrysogenum* using geneticin as a suitable transformation marker.. FEMS Microbiol Lett.

[29] Li X, Kim SK, Nam KW, Kang JS, Choi HD (2006). A new antibacterial dioxopiperazine alkaloid related to gliotoxin from a marine isolate of the fungus Pseudallescheria.. J Antibiot (Tokyo).

[30] Losada L, Ajayi O, Frisvad JC, Yu J, Nierman WC (2009). Effect of competition on the production and activity of secondary metabolites in Aspergillus species.. Med Mycol.

[31] Watanabe A, Kamei K, Sekine T, Waku M, Nishimura K (2004). Effect of aeration on gliotoxin production by *Aspergillus fumigatus* in its culture filtrate.. Mycopathologia.

[32] Pontecorvo G, Rroper JA, Hemmons LM, MacDonald KD, Bufton AW (1953). The genetics of *Aspergillus nidulans.*. Adv Genet.

[33] Punt PJ, Oliver RP, Dingemanse MA, Pouwels PH, van den Hondel CA (1987). Transformation of Aspergillus based on the hygromycin B resistance marker from *Escherichia coli*.. Gene.

[34] Langfelder K, Philippe B, Jahn B, Latgé JP, Brakhage AA (2001). Differential expression of the *Aspergillus fumigatus pksP* gene detected in vitro and in vivo with green fluorescent protein.. Infect Immun.

[35] Tilburn J, Sánchez-Ferrero JC, Reoyo E, Arst HN, Peñalva MA (2005). Mutational analysis of the pH signal transduction component PalC of *Aspergillus nidulans* supports distant similarity to BRO1 domain family members.. Genetics.

[36] Carberry S, Neville CM, Kavanagh KA, Doyle S (2006). Analysis of major intracellular proteins of Aspergillus fumigatus by MALDI mass spectrometry: identification and characterisation of an elongation factor 1B protein with glutathione transferase activity.. Biochem Biophys Res Commun.

[37] Reeves EP, Messina CG, Doyle S, Kavanagh K (2004). Correlation between gliotoxin production and virulence of *Aspergillus fumigatus* in *Galleria mellonella.*. Mycopathologia.

[38] Bradford MM (1976). A rapid and sensitive method for the quantitation of microgram quantities of protein utilizing the principle of protein-dye binding.. Anal Biochem.

[39] Hill KE, McCollum GW, Burk RF (1997). Determination of thioredoxin reductase activity in rat liver supernatant.. Anal Biochem.

[40] Spellig T, Bottin A, Kahmann R (1996). Green fluorescent protein (GFP) as a new vital marker in the phytopathogenic fungus *Ustilago maydis*.. Mol Gen Genet.

[41] Loovers HM, Guinan E, Jones GW (2007). Importance of Hsp70 ATPase domain in prion propagation.. Genetics.

[42] Schwimmer C, Masison, DC (2002). Antagonistic interactions between yeast [*PSI*
^+^] and [*URE3*] prions and curing of [*URE3*] by Hsp70 protein chaperone Ssa1p but not by Ssa2p.. Mol Cell Biol.

[43] Hearn VM, Mackenzie DW (1980). Mycelial antigens from two strains of *Aspergillus fumigatus*: an analysis by two-dimensional immunoelectrophoresis.. Mykosen.

[44] Taylor JJ, Burroughs EJ (1973). Experimental avian aspergillosis.. Mycopathol Mycol Appl.

[45] Oberegger H, Eisendle M, Schrettl M, Graessle S, Haas H (2003). 4′-phosphopantetheinyl transferase-encoding *npgA* is essential for siderophore biosynthesis in *Aspergillus nidulans.*. Curr Genet.

