# Supplementary material for: Self-Protection against Gliotoxin—A Component of the Gliotoxin Biosynthetic Cluster, GliT, Completely Protects Aspergillus fumigatus Against Exogenous Gliotoxin
Source: PLoS Pathog. 2010 Jun 10;6(6):e1000952. doi: 10.1371/journal.ppat.1000952 (PMC2883607; doi:10.1371/journal.ppat.1000952)
Supplement: Protocol S1 — Supplementary data. (0.03 MB DOC) [file ppat.1000952.s001.doc]

**SUPPLEMENTARY DATA**

**Methods**

**Recombinant GliT purification.** For GliT purification, cells were lysed with lysozyme (90 μg/ml) and sodium deoxycholate (0.04 %(w/v)), in the presence of protease inhibitors (1 μg/ml leupeptin and pepstatin, respectively and 1 mM PMSF). Cell debris was removed by centrifugation at 10,000 *g* for 10 min. GliT was purified by differential extraction and N-terminal (His)_6_-tagged recombinant protein was purified from the supernatant by Ni-NTA chromatography (Qiagen, West Sussex, UK) by elution with 250 mM imidazole in 50 mM sodium phosphate/300 mM NaCl. Purified GliT was dialysed (three times; once overnight, and twice for 4 hours) against sodium carbonate containing 0.02% (w/v) sodium azide for storage at 4 ^o^C.

**Purification of native GliT from *A. fumigatus* by IEX.** *A. fumigatus* ATCC46645 mycelia were ground in liquid nitrogen and lysed in ice-cold lysis buffer as described (33) following incubaton with gliotoxin (10 μg/ml) for 3 h). Following centrifugation (12,000 *g*; 30 min), the lysate supernatant (176 ml) was ammonium sulphate precipitated (10, 20, 50 and 70 % ammonium sulphate). The 50 % pellet was resuspended in 20 mM Bis-Tris propane pH 7.6 and dialysed three times against 50 volumes of the same buffer at 4°C. The dialysate was centrifuged (12,000 *g*; 20 min) and filtered (0.45 μm) to remove particulates. The dialysate was loaded onto an equilibrated Q-Sepharose column (4 ml) at a flow rate of 1 ml/min. The column was washed with 20 mM Bis-Tris propane pH 7.6 before bound protein was eluted using an NaCl gradient (0.5 M final). Absorbance detection was at 280 nm and 454 nm. Collected fractions were subjected to SDS-PAGE, Western blot and activity analysis for GliT.
