# Supplementary material for: Self-Protection against Gliotoxin—A Component of the Gliotoxin Biosynthetic Cluster, GliT, Completely Protects Aspergillus fumigatus Against Exogenous Gliotoxin
Source: PLoS Pathog. 2010 Jun 10;6(6):e1000952. doi: 10.1371/journal.ppat.1000952 (PMC2883607; doi:10.1371/journal.ppat.1000952)
Supplement: Figure S1 — (A) Deletion of gliT and gliH in A. fumigatus ATCC46645 and 26933, respectively. Southern analysis of ΔgliT mutant versus wild-type DNA for A. fumigatus ATCC46645 and ATCC26933, respectively. Here, a DIG-labelled probe was used to detect the predicted presence of 3.3 and 6.4 kb fragments in XbaI restricted ΔgliT and wild-type DNA, respectively. (B) Southern Blot analysis of ΔgliT complemented strains (gliTC). Genomic DNA of wild-type and complemented strains was digested with NarI (ATCC46645) and ApaI (ATCC26933), respectively and probed using a DIG-labelled probe amplified using oligos ogliT-4 and ogliT-5. (C) Southern Blot analysis of ΔgliH, ΔgliH-complemented strains (gliHC) and ΔgliT-complemented with gliH (ΔgliT26933gliH). Genomic DNA of wild-type and respective mutant strains was digested with NdeI (ATCC26933) and probed using a DIG-labelled probe amplified using oligos ogliH-4 and ogliH-5. (1) A. fumigatus ATCC26993, (2) ΔgliH, (3) gliHC, (4) ΔgliT, (5) ΔgliT26933gliH. (1.30 MB DOC) [file ppat.1000952.s002.doc]

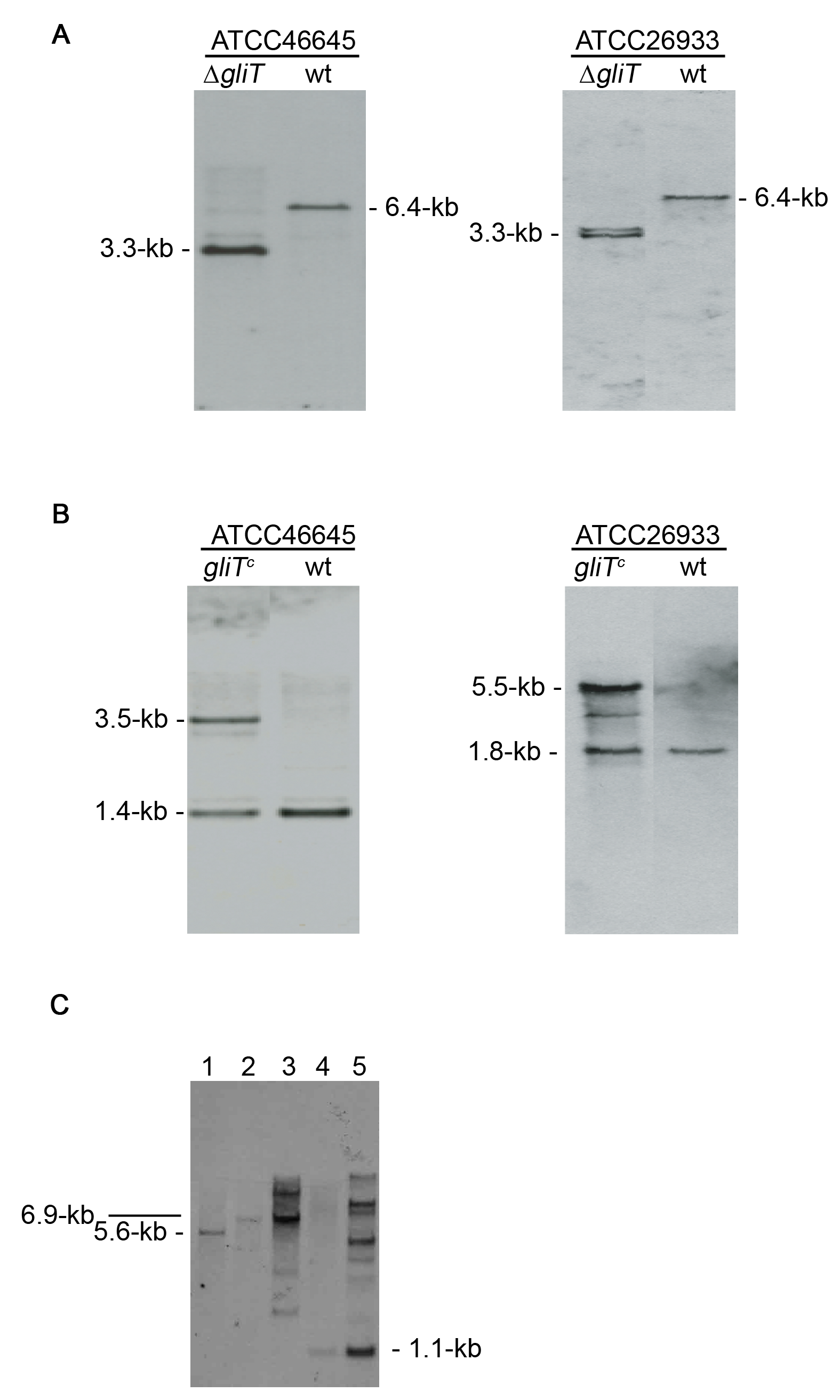


**Figure S1.** (A) Deletion of *gliT* and *gliH* in *A. fumigatus* ATCC46645 and 26933, respectively. Southern analysis of Δ*gliT* mutant versus wild-type DNA for *A. fumigatus* ATCC46645 and ATCC26933, respectively. Here, a DIG-labelled probe was used to detect the predicted presence of 3.3 and 6.4 kb fragments in *Xba*I restricted Δ*gliT* and wild-type DNA, respectively. (B) Southern Blot analysis of ∆*gliT* complemented strains (*gliT^C^*). Genomic DNA of wild-type and complemented strains was digested with *Nar*I (ATCC46645) and *Apa*I (ATCC26933), respectively and probed using a DIG-labelled probe amplified using oligos ogliT-4 and ogliT-5. (C) Southern Blot analysis of Δ*gliH*, ∆*gliH*-complemented strains (*gliH^C^*) and ∆*gliT*-complemented with *gliH* (∆*gliT*^26933^*^gliH^*). Genomic DNA of wild-type and respective mutant strains was digested with *Nde*I (ATCC26933) and probed using a DIG-labelled probe amplified using oligos ogliH-4 and ogliH-5. (1) *A. fumigatus* ATCC26933, (2) Δg*liH*, (3) *gliH^C^* , (4) ∆*gliT*, (5) *∆gliT*^26933^*^gliH^*.
