# Supplementary material for: Self-Protection against Gliotoxin—A Component of the Gliotoxin Biosynthetic Cluster, GliT, Completely Protects Aspergillus fumigatus Against Exogenous Gliotoxin
Source: PLoS Pathog. 2010 Jun 10;6(6):e1000952. doi: 10.1371/journal.ppat.1000952 (PMC2883607; doi:10.1371/journal.ppat.1000952)
Supplement: Figure S2 — Phenotypic analysis of A. fumigatus ATCC46645 (wild-type) and ΔgliT strains in the presence of gliotoxin (GT). Compared to wild-type, gliotoxin (5 µg/ml) significantly inhibits ΔgliT growth in minimal medium (MM) and completely inhibits ΔgliT growth in both MM and Sabouraud medium (10 µg/ml). (0.04 MB DOC) [file ppat.1000952.s003.doc]

**Figure S2.** Phenotypic analysis of *A. fumigatus* ATCC46645 (wild-type) and Δ*gliT* strains in the presence of gliotoxin (GT). Compared to wild-type, gliotoxin (5 μg/ml) significantly inhibits Δ*gliT* growth in minimal medium (MM) and completely inhibits Δ*gliT* growth in both MM and Sabouraud medium (10 μg/ml).
