# Supplementary material for: Self-Protection against Gliotoxin—A Component of the Gliotoxin Biosynthetic Cluster, GliT, Completely Protects Aspergillus fumigatus Against Exogenous Gliotoxin
Source: PLoS Pathog. 2010 Jun 10;6(6):e1000952. doi: 10.1371/journal.ppat.1000952 (PMC2883607; doi:10.1371/journal.ppat.1000952)

**Figure S3.** Peptide mass spectrum of GliT from *A. fumigatus* ATCC26933, a component of the gliotoxin biosynthetic cluster (33% sequence coverage). This MALDI-ToF identification represents the first proteomic confirmation of the expression of a component of the gliotoxin biosynthetic cluster.


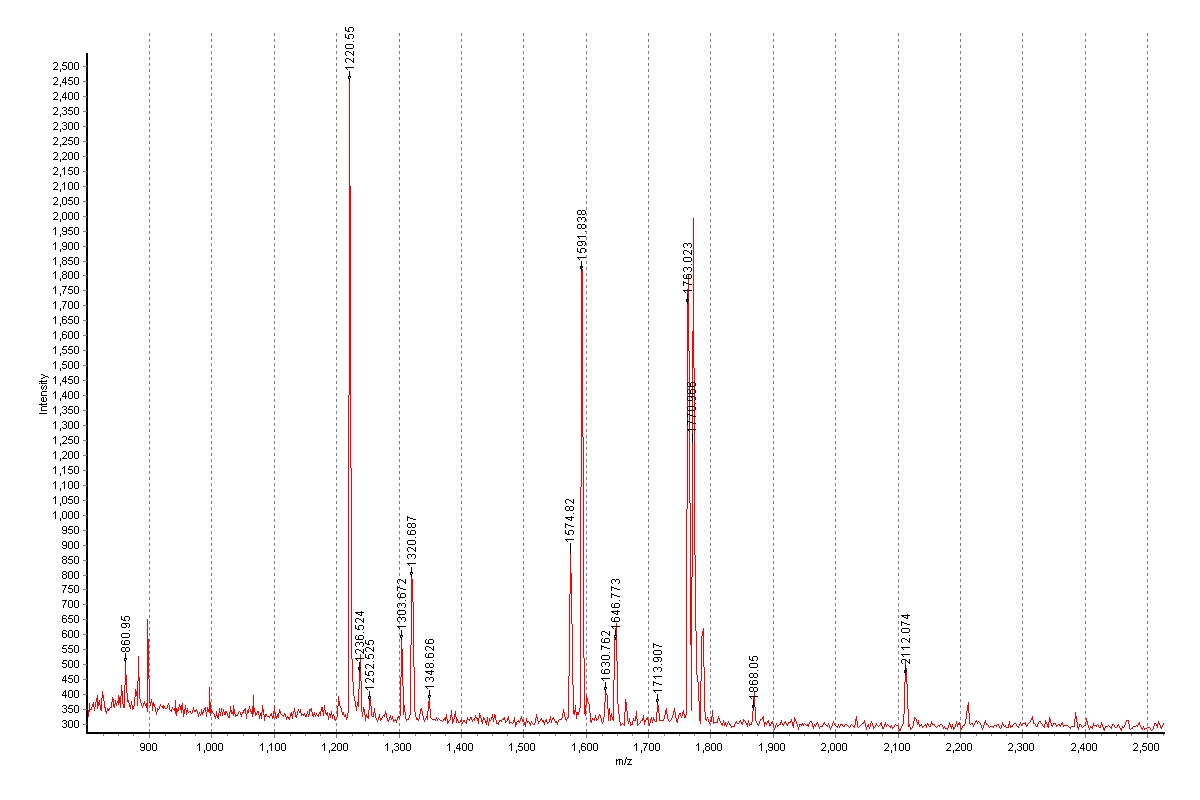

Supplement: Figure S3 — Peptide mass spectrum of GliT from A. fumigatus ATCC26933, a component of the gliotoxin biosynthetic cluster (33% sequence coverage). This MALDI-ToF identification represents the first proteomic confirmation of the expression of a component of the gliotoxin biosynthetic cluster. (0.11 MB DOC) [file ppat.1000952.s004.doc]
