# Supplementary material for: Self-Protection against Gliotoxin—A Component of the Gliotoxin Biosynthetic Cluster, GliT, Completely Protects Aspergillus fumigatus Against Exogenous Gliotoxin
Source: PLoS Pathog. 2010 Jun 10;6(6):e1000952. doi: 10.1371/journal.ppat.1000952 (PMC2883607; doi:10.1371/journal.ppat.1000952)
Supplement: Figure S4 — Analysis of gliotoxin, and related metabolite, production in A. fumigatus mutant strains. (A) Gliotoxin was detectable by RP-HPLC (data not shown) and LC-MS in A. fumigatus ATCC26933 gliT c with identical molecular mass and fragmentation pattern to commercially available gliotoxin and as reported in [10]. (B) LC-ToF analysis of RP-HPLC purified gliotoxin-related metabolite (Figure 3B) from Aspergillus fumigatus ΔgliT 26933. MS spectrum shows the presence of a high abundance molecular ion (Retention time = 9.153 min) with m/z 279.0796 (M+H)+ (557.1497 (2M+H)+) which corresponds precisely to a predicted molecular formula of C13 H14 N2 O3 S - a putative monothiol form of gliotoxin. (C) LC-MS analysis analysis of RP-HPLC purified gliotoxin-related metabolite (Figure 3B) from Aspergillus fumigatus ΔgliT 26933. Using a manual approach, LC-MS software identified five molecular species with m/z 279.0. The most intense peaks (1 and 5) were subjected to MS2 analysis and both yielded identical fragments ions of m/z 261.1, 231.0 and 203.1. Notably, peak 1 eluted from LC-MS and LC-ToF with an identical retention time (9.1 min) (D) Gliotoxin production was undetectable in A. fumigatus ΔgliH26933, by RP-HPLC and LC-MS (data not shown), thereby indicating a role for this gene in either gliotoxin biosynthesis or secretion. (0.43 MB DOC) [file ppat.1000952.s005.doc]

**A**

**
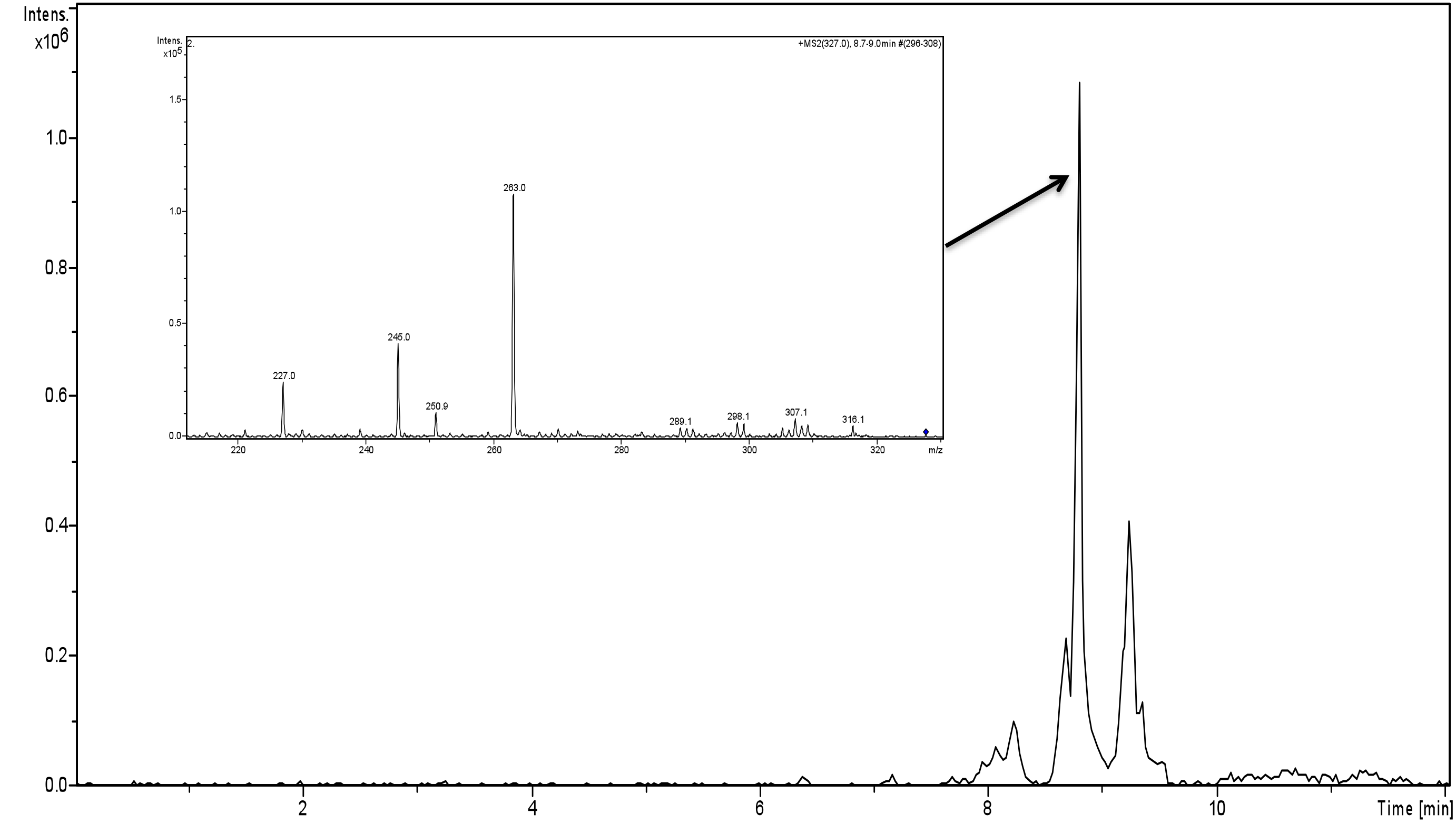
**

**B**

**
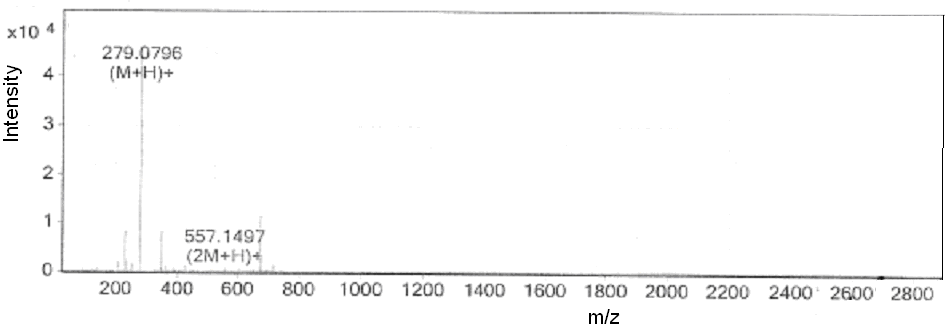
**

**C**


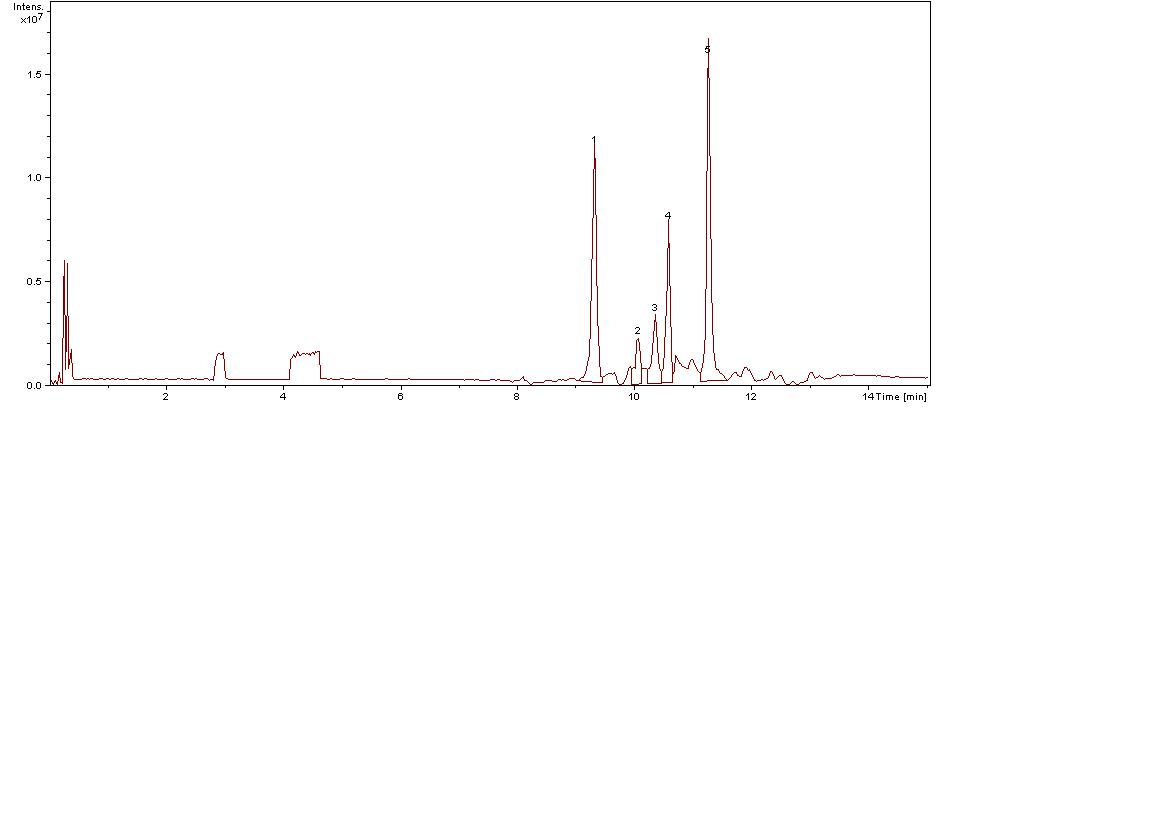


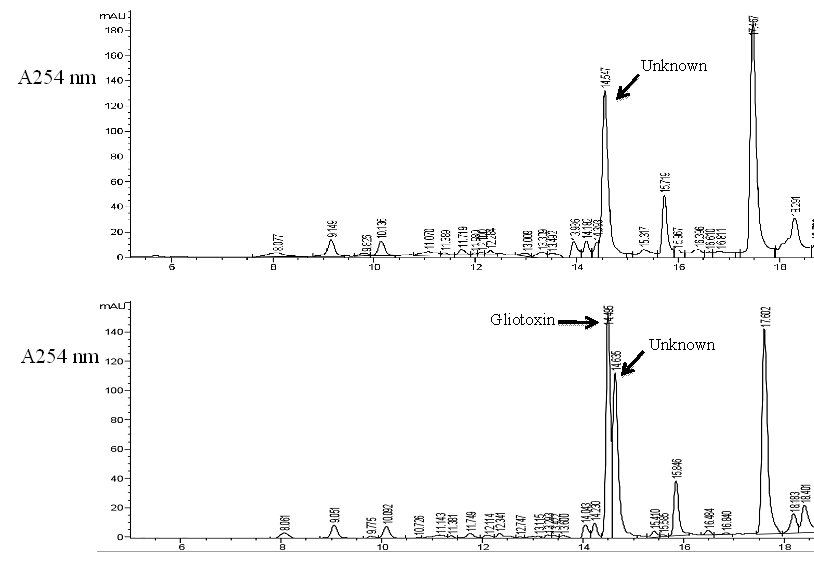


**D**

**Figure S4.** Analysis of gliotoxin, and related metabolite, production in *A. fumigatus* mutant strains. (A) Gliotoxin was detectable by RP-HPLC (data not shown) and LC-MS in *A. fumigatus* ATCC26933 *gliT*^c^ with identical molecular mass and fragmentation pattern to commercially available gliotoxin and as reported in [10]. (B) LC-ToF analysis of RP-HPLC purified gliotoxin-related metabolite (Figure 3B) from *Aspergillus fumigatus* Δ*gliT*^26933^. MS spectrum shows the presence of a high abundance molecular ion (Retention time = 9.153 min) with m/z 279.0796 (M+H)^+^ (557.1497 (2M+H)^+^) which corresponds precisely to a predicted molecular formula of C13 H14 N2 O3 S - a putative monothiol form of gliotoxin. (C) LC-MS analysis analysis of RP-HPLC purified gliotoxin-related metabolite (Figure 3B) from *Aspergillus fumigatus* Δ*gliT*^26933^. Using a manual approach, LC-MS software identified five molecular species with m/z 279.0. The most intense peaks (1 and 5) were subjected to MS^2^ analysis and both yielded identical fragments ions of m/z 261.1, 231.0 and 203.1. Notably, peak 1 eluted from LC-MS and LC-ToF with an identical retention time (9.1 min) (D) Gliotoxin production was undetectable in *A. fumigatus* Δ*gliH*^26933^, by RP-HPLC and LC-MS (data not shown), thereby indicating a role for this gene in either gliotoxin biosynthesis or secretion.
