# Supplementary material for: Self-Protection against Gliotoxin—A Component of the Gliotoxin Biosynthetic Cluster, GliT, Completely Protects Aspergillus fumigatus Against Exogenous Gliotoxin
Source: PLoS Pathog. 2010 Jun 10;6(6):e1000952. doi: 10.1371/journal.ppat.1000952 (PMC2883607; doi:10.1371/journal.ppat.1000952)
Supplement: Figure S5 — Recombinant GliT expression. (A) SDS-PAGE and (B) Western blot analysis of recombinant GliT expression and solubility. Lane 1 contains non-transformed BL21 (DE3) cells. Lane 2 contains non-induced cell extract and lanes 3–5 contain induced cell extracts taken 1–3 h post-induction with 0.6 mM IPTG. Lane 6 and 7 contain soluble and insoluble cell extracts respectively. Lane 8 contains His-tag positive control and lane 9 contains non-reducing cell extract- monomeric (m) and dimeric (d) forms of GliT are evident. Lane M contains molecular mass marker. (2.29 MB DOC) [file ppat.1000952.s006.doc]

**A**

1 2 3 4 5 6 7 8 9 M

kDa

175

82

63

47.5

32.5


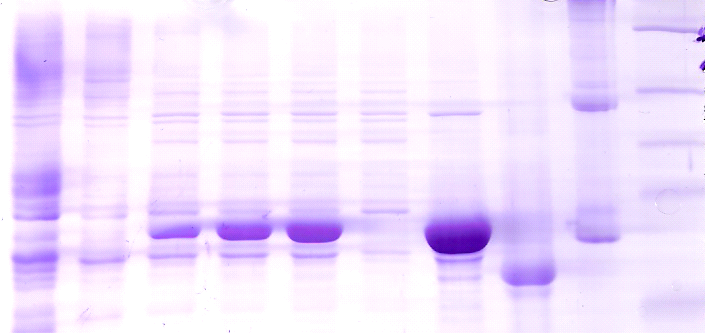


kDa

47.5

32.5

GliT

GliT (m)

GliT (d)

**B**

kDa

175

82

63

47.5

32.5

1 2 3 4 5 6 7 8 9 M


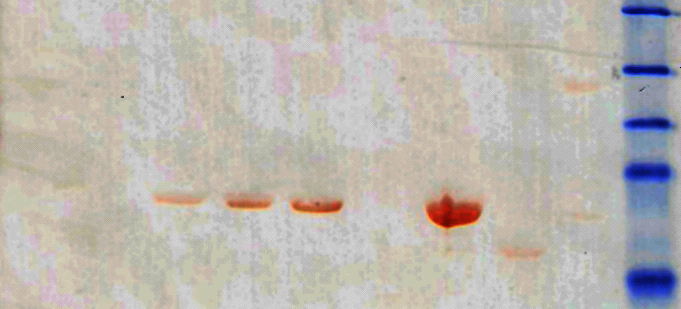


GliT (m)

GliT (d)

**Figure S5.** Recombinant GliT expression. (A) SDS-PAGE and (B) Western blot analysis of recombinant GliT expression and solubility. Lane 1 contains non-transformed BL21 (DE3) cells. Lane 2 contains non-induced cell extract and lanes 3-5 contain induced cell extracts taken 1-3 h post-induction with 0.6 mM IPTG. Lane 6 and 7 contain soluble and insoluble cell extracts respectively. Lane 8 contains His-tag positive control and lane 9 contains non-reducing cell extract- monomeric (m) and dimeric (d) forms of GliT are evident. Lane M contains molecular mass marker.
