# Supplementary material for: Self-Protection against Gliotoxin—A Component of the Gliotoxin Biosynthetic Cluster, GliT, Completely Protects Aspergillus fumigatus Against Exogenous Gliotoxin
Source: PLoS Pathog. 2010 Jun 10;6(6):e1000952. doi: 10.1371/journal.ppat.1000952 (PMC2883607; doi:10.1371/journal.ppat.1000952)
Supplement: Figure S6 — Confirmation of recombinant GliT identity by MALDI-ToF mass spectrometry (21% sequence coverage). (0.03 MB DOC) [file ppat.1000952.s007.doc]

**Recombinant GliT:**

MSIGKLLSNGALLVDVLIIGAGPAGLSTATGLAR**QLHTAVVFDSGVYR**NAK**TQHMHNVLGWDHRNPAELR**AAGRADLTTR**YSTIQFQNSTIEAIR**QVETNQLFEAR**DNEGHSWYGRKVVLATGVR**DIPLDIEGYSECWANGIYHCLFCDGYEERGQETVGVLALGPIANPARALHLARMAHLGESESKTEGFLVYNPQTEVNGPFAKQLALNMTEGGDILTTPPFYETSVPGVFAVGDCATPLKAVTPAVSMGSLAAGGLVAQLQAQALPEFRLDQEL

**Figure S6.** Confirmation of recombinant GliT identity by MALDI-ToF mass spectrometry (21% sequence coverage).
