# Supplementary material for: Self-Protection against Gliotoxin—A Component of the Gliotoxin Biosynthetic Cluster, GliT, Completely Protects Aspergillus fumigatus Against Exogenous Gliotoxin
Source: PLoS Pathog. 2010 Jun 10;6(6):e1000952. doi: 10.1371/journal.ppat.1000952 (PMC2883607; doi:10.1371/journal.ppat.1000952)
Supplement: Figure S7 — (A) Partial purification and immunological identification of GliT. Absorbance (A280 nm and A454 nm) versus elution volume (ml) for a Q-Sepharose ion-exchange fractionation of GliT dialysate (post-ammonium sulphate precipitation). (B) SDS-PAGE analysis of Q-Sepharose ion-exchange chromatography (IEX) fractions. (C) Western blot analysis of Q-Sepharose IEX fractions using human IgG[anti-GliT] and anti-human IgG-HRP conjugate with ECL detection. These fractions were pooled and used for activity and immunological analysis as shown in Figures 4 and 5. (0.29 MB DOC) [file ppat.1000952.s008.doc]

**A**


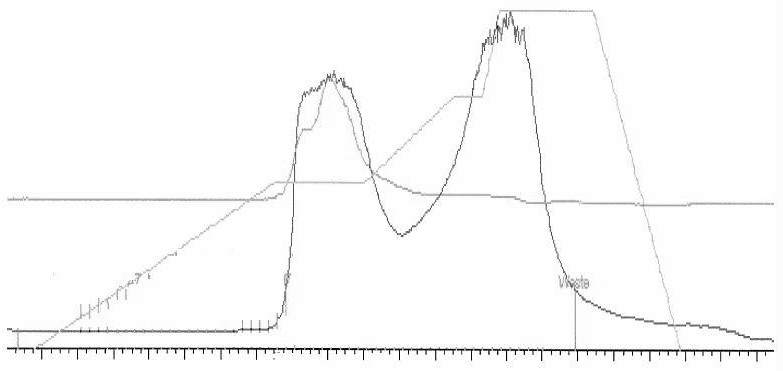


Absorbance

1 4 8 12 16 18 22 26 30 34 10 15 20 55

280 nm

454 nm

Elution volume (ml)

**B**


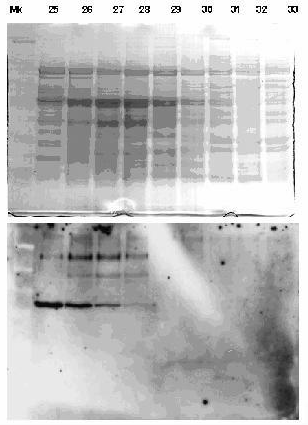


GliT


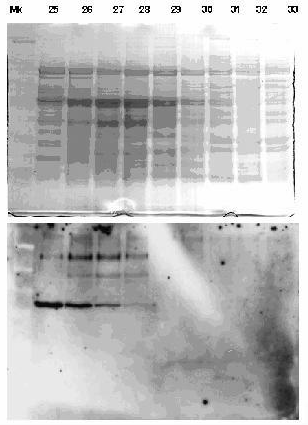


GliT

**C**

**Figure S7.** (A) Partial purification and immunological identification of GliT. Absorbance (A280 nm and A454 nm) versus elution volume (ml) for a Q-Sepharose ion-exchange fractionation of GliT dialysate (post-ammonium sulphate precipitation). (B) SDS-PAGE analysis of Q-Sepharose ion-exchange chromatography (IEX) fractions. (C) Western blot analysis of Q-Sepharose IEX fractions using human IgG[anti-GliT] and anti-human IgG-HRP conjugate with ECL detection. These fractions were pooled and used for activity and immunological analysis as shown in Figures 4 and 5.
