# Supplementary material for: Self-Protection against Gliotoxin—A Component of the Gliotoxin Biosynthetic Cluster, GliT, Completely Protects Aspergillus fumigatus Against Exogenous Gliotoxin
Source: PLoS Pathog. 2010 Jun 10;6(6):e1000952. doi: 10.1371/journal.ppat.1000952 (PMC2883607; doi:10.1371/journal.ppat.1000952)
Supplement: Figure S8 — Gliotoxin induces expression of GliT-GFP expression in A. fumigatus. (A) Basal expression of GliT-GFP expression, determined by confocal fluorescence microscopy in A. fumigatus in the absence of added gliotoxin. Panel I: GliT-GFP fluorescence, panel II: DAPI nuclear staining and panel III: Image merge. (B) Enhanced expression of GliT-GFP throughout mycelia following exposure to gliotoxin (5 µg/ml). Panel I: GliT-GFP fluorescence, panel II: DAPI nuclear staining and panel III: image merge. (C) Fluorescence intensity for DAPI (blue) and GFP (green) are shown. Yellow line corresponds to the fluorescence intensities depicted with a red arrow. Intensities demonstrate localisation of GliT-GFP in cytoplasm and nuclei. (1.42 MB DOC) [file ppat.1000952.s009.doc]

I II III


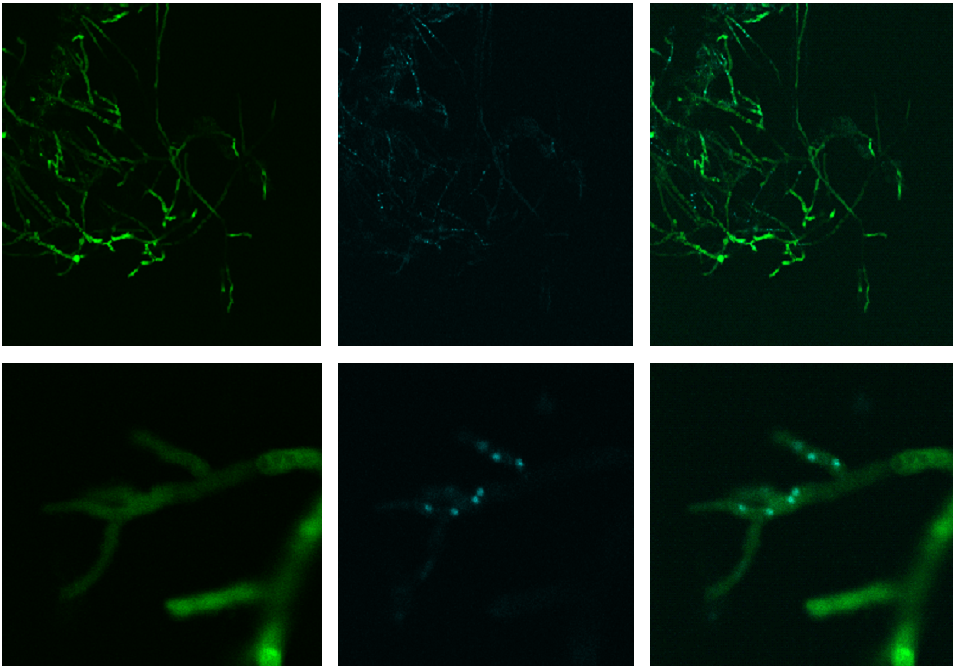


**A**

I II III
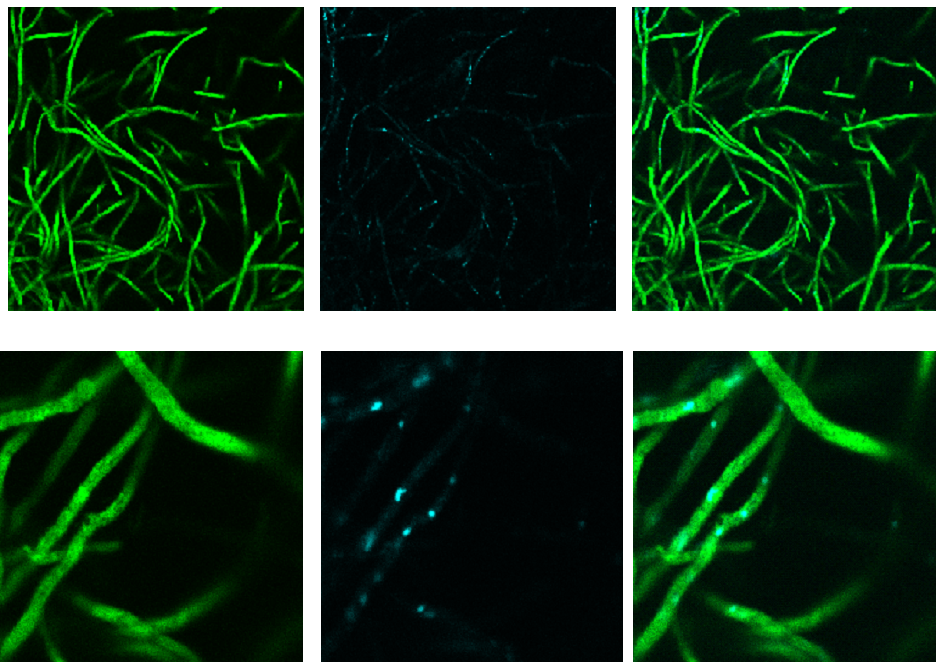


**B**

**C**

**
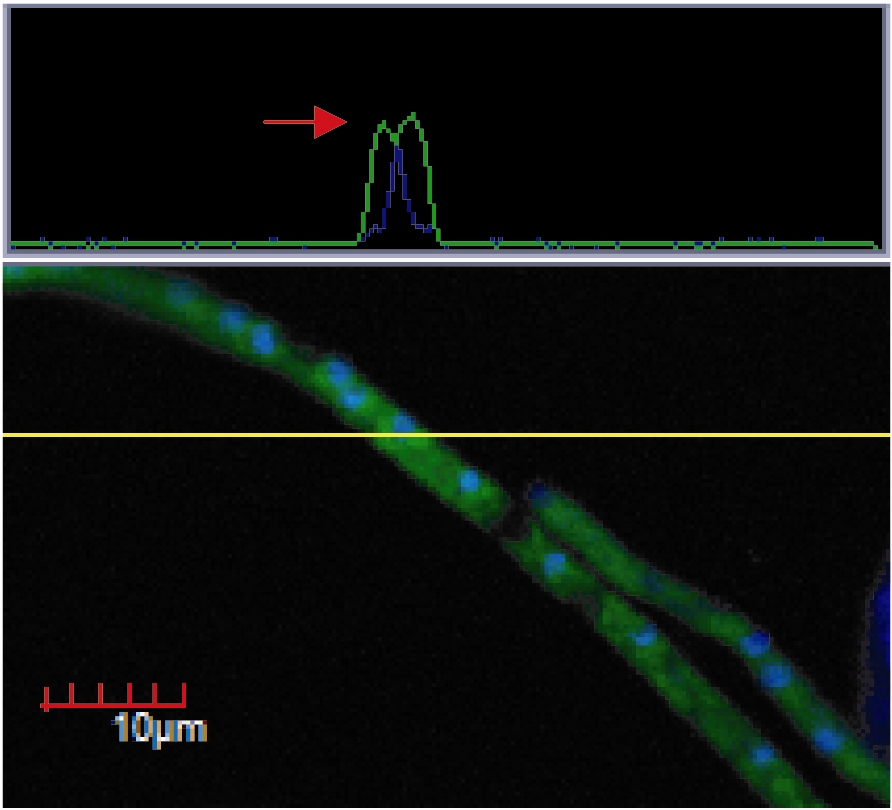
**

**Figure S8.** Gliotoxin induces expression of GliT-GFP expression in *A. fumigatus*. (A) Basal expression of GliT-GFP expression, determined by confocal fluorescence microscopy in *A. fumigatus* in the absence of added gliotoxin. Panel I: GliT-GFP fluorescence, panel II: DAPI nuclear staining and panel III: Image merge. (B) Enhanced expression of GliT-GFP throughout mycelia following exposure to gliotoxin (5 μg/ml). Panel I: GliT-GFP fluorescence, panel II: DAPI nuclear staining and panel III: image merge. (C) Fluorescence intensity for DAPI (blue) and GFP (green) are shown. Yellow line corresponds to the fluorescence intensities depicted with a red arrow. Intensities demonstrate localisation of GliT-GFP in cytoplasm and nuclei.
