# Supplementary material for: Self-Protection against Gliotoxin—A Component of the Gliotoxin Biosynthetic Cluster, GliT, Completely Protects Aspergillus fumigatus Against Exogenous Gliotoxin
Source: PLoS Pathog. 2010 Jun 10;6(6):e1000952. doi: 10.1371/journal.ppat.1000952 (PMC2883607; doi:10.1371/journal.ppat.1000952)
Supplement: Figure S9 — Expression of GliT-GFP restores resistance to exogenous gliotoxin. Phenotypes of A. fumigatus ATCC46645 (WT), ΔgliT 46645, gliTC and gliTgfp. Conidia of the respective strain were point inoculated on AMM plates in the absence and presence of gliotoxin (5 and 10 µg/ml. respectively) and incubated for 40 h at 37°C. (0.62 MB DOC) [file ppat.1000952.s010.doc]

WT *∆gliT gliT^C^ gliT^gfp^*

**
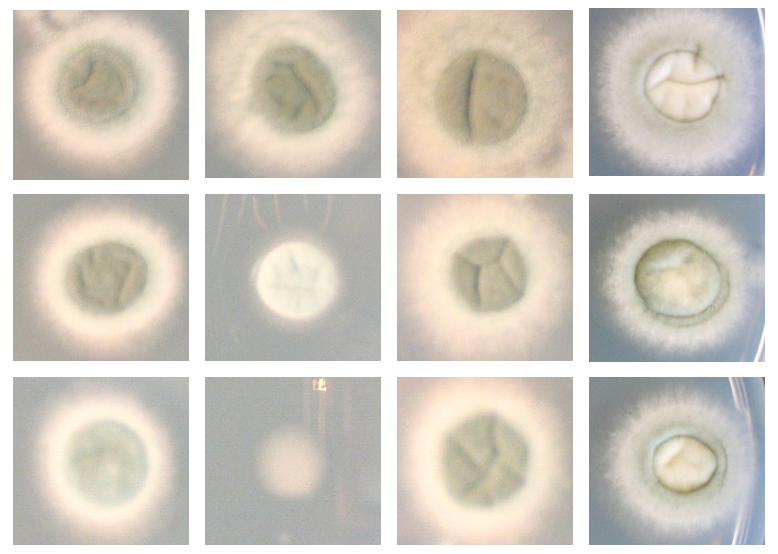
**

AMM

Only

Gliotoxin

(5 µg/ml)

Gliotoxin

(10 µg/ml)

**Figure S9.** Expression of GliT-GFP restores resistance to exogenous gliotoxin. Phenotypes of *A. fumigatus* ATCC46645 (WT), *∆gliT*^46645^, *gliT^C^* and *gliT^gfp^*. Conidia of the respective strain were point inoculated on AMM plates in the absence and presence of gliotoxin (5 and 10 μg/ml. respectively) and incubated for 40 h at 37 ºC.
