# Supplementary material for: Self-Protection against Gliotoxin—A Component of the Gliotoxin Biosynthetic Cluster, GliT, Completely Protects Aspergillus fumigatus Against Exogenous Gliotoxin
Source: PLoS Pathog. 2010 Jun 10;6(6):e1000952. doi: 10.1371/journal.ppat.1000952 (PMC2883607; doi:10.1371/journal.ppat.1000952)
Supplement: Figure S10 — Virulence assay of A. fumigatus wild-type, ΔgliZ, ΔgliT and gliTc. (A and B) G. mellonella challenged with A. fumigatus ΔgliZ [14], corresponding wild-type, ΔgliT 26933 and gliT c in the presence (A) (6 ng, pre-incubation 2 hr prior to conidial challenge) and absence (B) of gliotoxin. (0.24 MB DOC) [file ppat.1000952.s011.doc]

**A**

***G. mellonella* survival when challenged with *A. fumigatus* strains (gliotoxin present).**


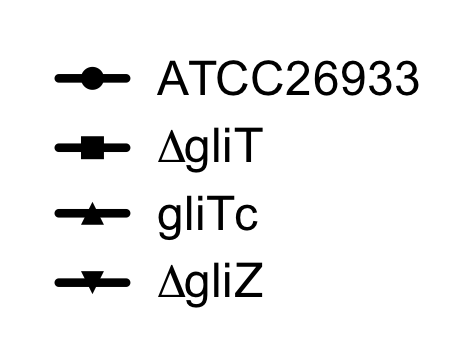


**wild-type**

**Δ*gliT***

***gliT*^c^**

**Δ*gliZ***


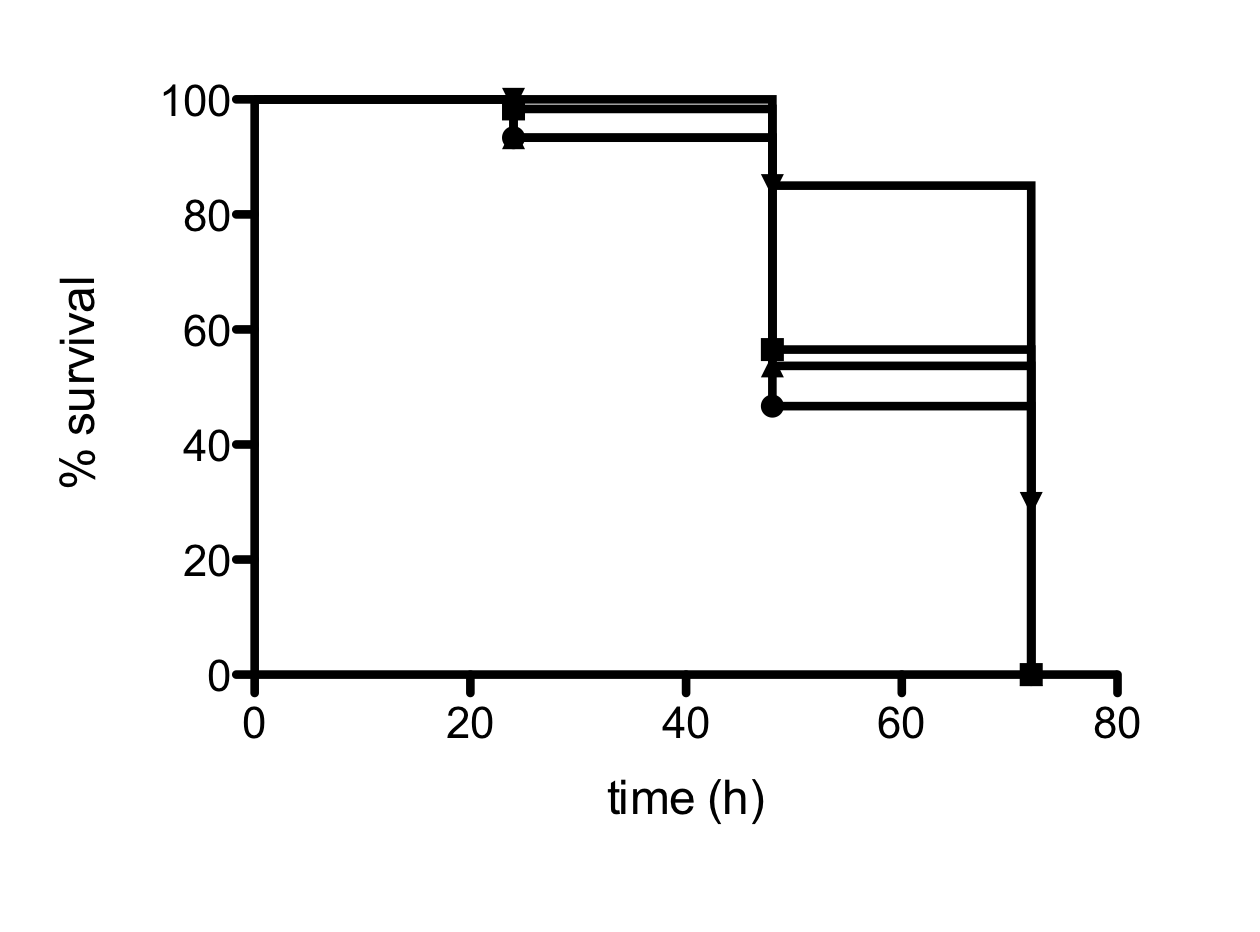


**B**

***G. mellonella* survival when challenged with *A. fumigatus* strains (gliotoxin absent).**


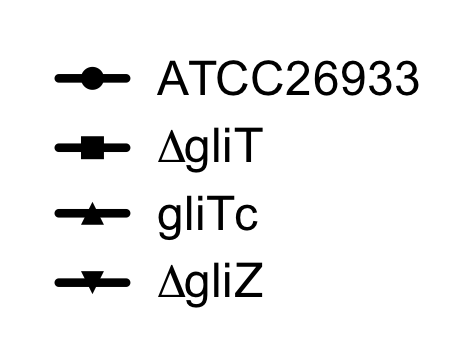


**wild-type**

**Δ*gliT***

***gliT*^c^**

**Δ*gliZ***


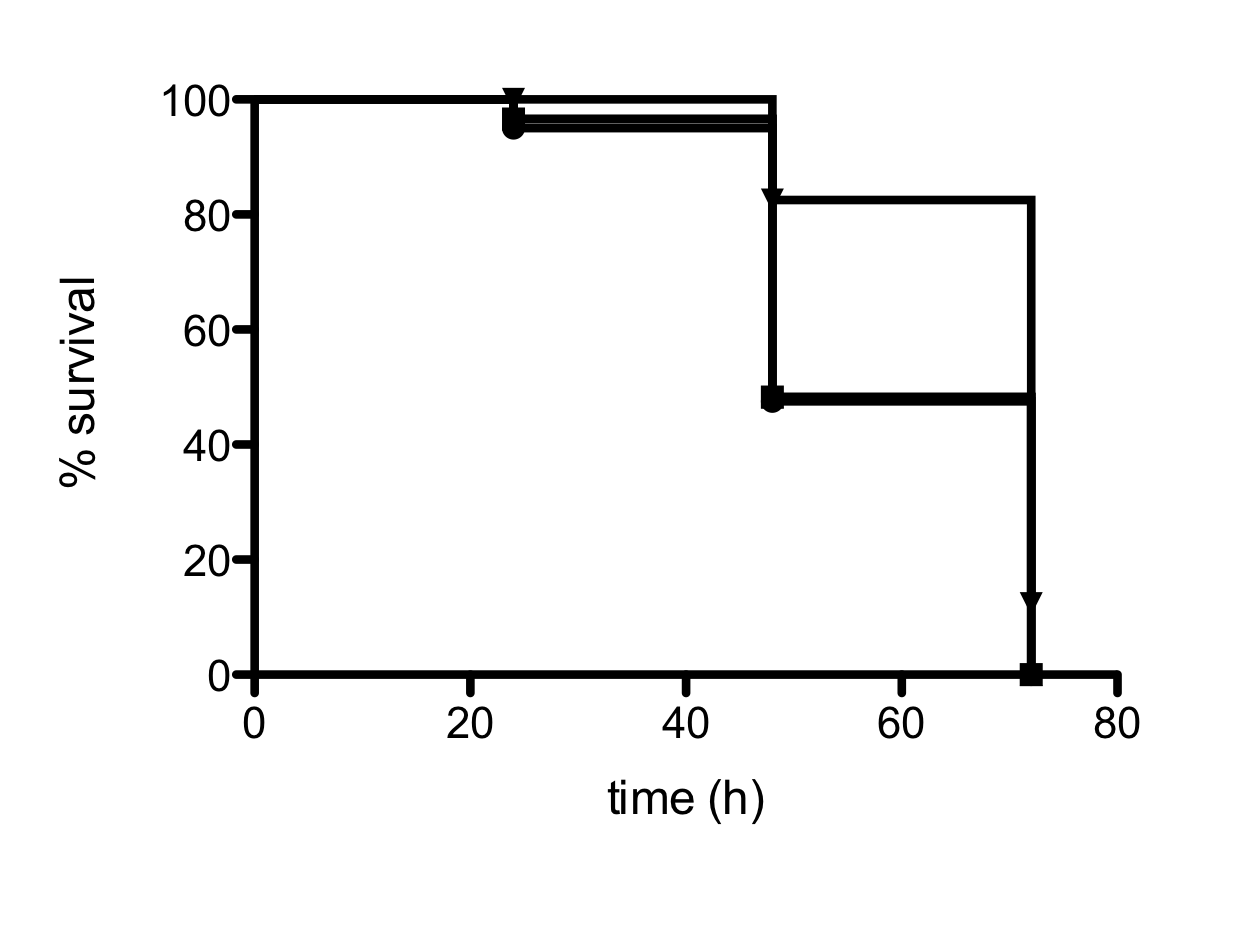


**Figure S10. Virulence assay of *A. fumigatus* wild-type, ∆*gliZ*, ∆*gliT* and *gliT^c^*.** (A and B) *G. mellonella* challenged with *A. fumigatus* **∆***gliZ* [14]*,* corresponding wild-type, ∆*gliT*^26933^ and *gliT*^c^ in the presence (A) (6 ng, pre-incubation 2 hr prior to conidial challenge) and absence (B) of gliotoxin.
